# Conversion of boreal forests to agricultural systems: soil microbial responses along a land-conversion chronosequence

**DOI:** 10.1186/s40793-024-00576-3

**Published:** 2024-05-11

**Authors:** Paul Benalcazar, Brent Seuradge, Amanda C. Diochon, Randall K. Kolka, Lori A. Phillips

**Affiliations:** 1 Faculty of Natural Resources Management, Lakehead University, Thunder Bay, ON Canada; 2 Agriculture and Agri-Food Canada, Harrow Research and Development Centre, Harrow, ON Canada; 3 Department of Geology, Lakehead University, Thunder Bay, ON Canada; 4 USDA Forest Services Northern Research Station, Grand Rapid, MN 55744 USA

**Keywords:** Climate change, Northern agricultural expansion, Soil microbiome, Biogeochemical cycling, Functional genes, Amplicon sequencing

## Abstract

**Background:**

Boreal regions are warming at more than double the global average, creating opportunities for the northward expansion of agriculture. Expanding agricultural production in these regions will involve the conversion of boreal forests to agricultural fields, with cumulative impacts on soil microbial communities and associated biogeochemical cycling processes. Understanding the magnitude or rate of change that will occur with these biological processes will provide information that will enable these regions to be developed in a more sustainable manner, including managing carbon and nitrogen losses. This study, based in the southern boreal region of Canada where agricultural expansion has been occurring for decades, used a paired forest-adjacent agricultural field approach to quantify how soil microbial communities and functions were altered at three different stages post-conversion (< 10, > 10 and < 50, and > 50 years). Soil microbial functional capacity was assessed by quantitative PCR of genes associated with carbon (C), nitrogen, and phosphorous (P) cycling; microbial taxonomic diversity and community structure was assessed by amplicon sequencing.

**Results:**

Fungal alpha diversity did not change, but communities shifted from Basidiomycota to Ascomycota dominant within the first decade. Bacterial alpha diversity increased, with Gemmatimonadota groups generally increasing and Actinomycetota groups generally decreasing in agricultural soils. These altered communities led to altered functional capacity. Functional genes associated with nitrification and low molecular weight C cycling potential increased after conversion, while those associated with organic P mineralization potential decreased. Stable increases in most N cycling functions occurred within the first decade, but C cycling functions were still changing 50 years post conversion.

**Conclusions:**

Microbial communities underwent a rapid shift in the first decade, followed by several decades of slower transition until stabilizing 50 years post conversion. Understanding how the microbial communities respond at different stages post-conversion improves our ability to predict C and N losses from emerging boreal agricultural systems, and provides insight into how best to manage these soils in a way that is sustainable at the local level and within a global context.

**Supplementary Information:**

The online version contains supplementary material available at 10.1186/s40793-024-00576-3.

## Introduction

Northern regions are warming at more than double the global average, with the growing season lengthening by an average of two days per decade since the 1950’s [1]. These climate change driven shifts in temperature and precipitation patterns are accelerating land use changes, including the northward expansion of agriculture [2, 3]. Current models predict that, on average, 76% of boreal regions will have sufficient growing degree days to accommodate crop production systems by the end of the 21st century [2]. Expanding agricultural production in most of these regions will require the clearing of boreal forests, which typically involves clear-cutting trees then bulldozing and burning stumps and all other vegetation [1, 4]. When forests are converted to cropping systems soil compaction, erosion, and salinization typically increase, and soil organic matter typically decreases [5–7]. In short, soils become degraded relative to their original state and their overall health and function is altered [8]. These cumulative impacts alter carbon (C), nitrogen (N), and phosphorus (P) cycling in the converted soils [9–11], including pathways associated with C- and N-based greenhouse gas (GHG) emissions [2, 12]. The magnitude of change in these processes after conversion will be influenced by soil microbial community responses and, in particular, those groups that underpin biogeochemical cycling processes [13–15]. In order to develop northern regions in the most sustainable manner possible, we need a better understanding of the direction, rate, and extent of change that will occur with these functional groups after land conversion.

A global meta-analysis of forest disturbances found that land conversion from forest to agriculture decreased overall bacterial and fungal abundance, with total microbial carbon and nitrogen decreasing up to 56.7% and 54.5% (respectively), but increased both bacterial and fungal diversity [16]. Within this meta-analysis however, the only boreal site examined microbial responses to forest age and not to agricultural conversion [17]. A recent study in northern boreal regions in Canada [10] found that bacterial abundance was not impacted by land conversion to either grassland or cropland, while archaeal abundance increased and fungal abundance decreased. Regardless of the direction of change, any shift in microbial community structure will lead to altered microbial functionality, due in part to an altered abundance of taxa important for both C (e.g. Actinomycetota, Gemmatimonadota, Bacteroidota) and N cycling (e.g. Pseudomonadota, Bacillota, Nitrospirota) [7, 18, 19]. The Zhou et al. [16] meta-analysis found that forest conversion generally decreased the relative abundance of Actinomycetota, but overall impacts on Pseudomonadota and Bacteroidota varied according to the intensity of land use change.

The type of imposed agricultural management practice, in addition to land use change itself, will also alter microbial communities and their functional capacity [18, 20, 21]. The addition of organic amendments such as manures or composts may increase both fungal and bacterial abundance [18, 21, 22]. Increased inputs of mineral fertilizers however, may increase bacterial but not fungal growth, as was found with N and P inputs in European sub-arctic systems [23, 24]. Shifts in bacterial populations under fertilization will be associated with direct changes in C and N cycling capacity [25]. When forests in Amazon regions were converted for agriculture, for example, microbial nitrification capacity increased by an order of magnitude [18]. Most, if not all, converted sites will also undergo tillage, which typically leads to increased rates of organic matter decomposition and subsequent nutrient release [26]. During forest conversion, the combination of tillage and generally altered carbon use efficiency associated with shifts in pH and nutrient balances [10, 16] could accelerate total C loss rates. Conversely, N addition could increase microbial metabolic efficiency and promote the formation of stable mineral-associated soil organic carbon [9, 10, 27]. Although the overall outcome in any given region will be influenced by microbial communities and competition for available nutrients [24], both C and N loss rates are likely to be high in the initial years following conversion until soil communities equilibrate and a new stable state is reached [28].

Few studies have evaluated how microbial nutrient cycling processes are altered following land conversion in northern regions, in part because these types of land use changes were relatively rare in the past. Boreal forest soils in Alaska, USA [29, 30], and Estonia [31] are known to have a high denitrifying capacity, which could be a significant N loss pathway following conversion. Seitz et al. [30] examined changes in diversity and associated functionality when vegetation and/or organic layers were removed from a permafrost site in Alaska. The researchers found that disturbance increased denitrification (nitrate reductase) and ligno-cellulose decomposition functions, and also increased the abundance of nitrifying taxa (Nitrosomonadaceae). Clearcutting in other northern forested systems has also been shown to increase nitrifier abundance [32], potentially due to a reduction in the deposition of volatile monoterpenes from conifers [33]. Boreal forest conversion for agricultural purposes, which will include both clear-cutting and the addition of fertilizer N, is likely to increase both nitrification and denitrification processes.

Climate change has expanded the northern boundaries of food production; however, a comprehensive understanding of how land conversion to agriculture in these regions will alter soil microbial communities and their biogeochemical cycling functions is currently incomplete and poorly understood [34]. Understanding how soil microbial systems are altered by land conversion at different stages post-conversion will provide information on how best to manage these expanding agricultural regions in a way that is sustainable at both the local level (e.g., enhanced fertility) and within a global context (e.g., reduced C and N losses). In this study, we investigated the soil microbiome at the current northern boundary of agriculture in Ontario Canada, using paired sites to compare baseline mature forest conditions with those of adjacent fields converted from forest to agriculture < 10 years ago, > 10 years ago but < 50 years ago, and > 50 years ago. Our objective was to evaluate post-land conversion changes in microbial functional potential, diversity, and community composition in a chronosequence according to time since conversion. We used qPCR to assess changes in microbial biogeochemical cycling functions (C, N, and P cycling) and amplicon sequencing to examine microbial community composition and diversity. We hypothesized that after land conversion (a) functions associated with N cycling would increase, (b) increased soil N and P fertility would lead to increased bacterial but decreased fungal abundance, and (c) overall diversity would increase due to a greater diversity of plants and plant litter inputs associated with agronomic cropping systems.

## Materials and methods

### Study sites and soil sampling

Soil samples were collected from dairy farms in the Thunder Bay, Ontario region within the Murillo and Slate River area (45° 31’ to 48° 17’ N, 89° 30’ to 89° 22’ W; Supplementary Figure S1). The Murillo and Slate River regions were formed from water-laid alluvial silt, sand, and gravel deposits that vary depending on the basin and depth [35]. Thunder Bay is part of the ecoregion 3 W (Lake Nipigon), located on the Precambrian Shield with substantial basalt and volcanic rock formations with low to moderate buffering capacity [36]. These soils are characterized by high water retention capacity, low permeability, and poor drainage [35]. Each farm included a mature mixed-wood forest and fields that have been cultivated for less than ten years (< 10 y), between ten and 50 years (> 10 and < 50 y), and/or more than 50 years (> 50 y). Converted sites have been under conventional management systems with tillage, synthetic fertilizers, spring and fall manure applications, and crop rotations of alfalfa, barley, corn, canola, and spring and winter wheat [8].

Soil samples were collected from 30 separate sites, encompassing cropped fields and adjacent forests, from July to August 2019 using a soil split-core sampler (AMS Soil Sampler, Inc., American Fall, Idaho). Surface plant litter was removed prior to sampling, which in the forest sites included the LF (litter-fermented) horizons. Within each site a sampling area of approximately 16 ha (∼500 m x 320 m) was defined and 5 plots (20 m x 20 m) were established at the center and corners of this sampling area. Three soil cores were randomly collected within each plot for a total of 15 cores per site (Supplementary Figure S1). Soil cores were separated into two depths (0–5 and 5–15 cm) and composited to provide 2 depth-related samples per site (60 final composite samples). Soil samples were kept cool (4 °C) during transport to the laboratory, where they were sieved (field-moist) through 8 mm sieves to remove plant and other large material, and then through 4 mm sieves to homogenize the composite samples. Sub-samples of the field-moist sieved soils were immediately frozen at -40 °C for molecular analyses, transported frozen to the Agriculture and Agri-Food Canada (AAFC) Harrow Research and Development Center (Harrow, Ontario), and stored at -80 °C until analysis.

Soil physico-chemical properties were determined as previously described [8]. Briefly, available soil macro- and micronutrients (P, K, Mg, Fe, Mn, and Zn) were measured using inductively coupled plasma optical emission spectrometry analysis (SPECTRO Analytical Instrument Inc.) after extraction with a modified Morgan solution (pH 4.8). Soil pH was measured using a 1:1 soil:water ratio. Organic matter was determined by loss on ignition in a muffle furnace at 500 °C. Total N and total C were analyzed using a LECO CHN 628 Series total elemental analyzer (LECO Corporation, St. Joseph, Michigan).

### Quantiative PCR evaluation of microbial function and abundance

DNA was extracted in duplicate (2 × 0.25 g) from soil samples using Qiagen DNeasy PowerSoil kits (Qiagen Inc., Canada), with an added 10 min incubation at 65 °C before bead-beating. DNA quality was first visualized on a 1.4% agarose gel, then quantity and quality were further evaluated using spectrophotometry (NanoDrop One, ThermoScientific). Once extraction reproducibility was confirmed, replicate extractions for each soil sample were pooled and DNA was re-quantified using fluorometric approaches (Quant-iT™ dsDNA BR kits; Life Technologies) then normalized to an initial working concentration of 10 ng µL^− 1^. For qPCR analyses, DNA was further diluted to a working concentration of 3.0 ng µL^− 1^ and then re-quantified using a high-sensitivity fluorometric assay (Quant-iT™ dsDNA HS kits; Life Technologies, USA).

Soil microbial functional capacity was assessed by qPCR of 17 key genes associated with C, N, and P cycling (Supplementary Table S1). In addition, the relative abundances of bacteria, archaea, and fungi were assessed by qPCR of 16S rRNA and 18S rRNA gene regions. A complete list of assays and associated functions are provided in Supplementary Table S1. Assays were performed in triplicate using the Bio-Rad CFX384 Touch Real-Time PCR Detection System (Bio-Rad, USA) in 5 µl reaction volumes containing 3.0 ng DNA (functional genes) or 0.3 ng DNA (taxonomic genes), and 0.12 µg of UltraPure™ BSA (Life Technologies, USA), with primer concentrations, commercial master mixes, and run conditions as listed in Supplementary Table S1. Gene abundance was calculated with respect to a plasmid-based standard curve with a concentration range from 2 to 2 × 10^7^ gene copies µl^–1^ DNA. Reaction efficiency ranged from 85 to 100%. Plasmids were generated by amplifying soil DNA in 50 µL PCR reactions using the appropriate primers (Table S1) and GoTaq master mix (Promega, USA), subcloning the cleaned (QIAquick PCR Purification Kit, Qiagen Inc., USA) PCR products into competent TOP10F’ One Shot *E. coli* (Invitrogen, USA), and extracting plasmids from positive clones (QIAprep Spin Miniprep Kit, Qiagen Inc., USA). Plasmids were sequenced (AAC, University of Guelph) and analyzed by *blastn* to confirm plasmid insert identity.

### Microbial community sequencing

The amplicon sequencing strategy followed the approach developed under the Government of Canada’s Genomics Research and Development Initiative (GRDI), EcoBiomics Project [37]. The primers for bacteria and archaea target the V4-V5 hypervariable region of the 16 S rRNA gene (515FY/926R) [38, 39] and the primers for fungi target the ITS2 region (ITS9F/ITS4R) [40, 41]. A 10 ng µL^− 1^ aliquot of each composited DNA sample was shipped on ice to Genome Quebec for library preparation and paired-end sequencing (2 × 250 bp) on an Illumina NovaSeq6000 platform. Bioinformatic analysis was performed on the General Purpose Science Cluster in Dorval, Quebec [37] using a custom workflow as outlined in Pérez-Guzmán et al. [42] and available in the supplemental materials.

A total of 12,843,763 16S rRNA paired-end amplicon sequences were generated from 60 samples, with 32,013 operational taxonomic units (OTUs; 98% nucleotide identity) identified after quality filtering and clustering. A total of 14,478,951 ITS paired-end amplicon sequences were generated, with 16,082 OTUs (98% nucleotide identity) identified after quality filtering and clustering. Detailed information about sequencing QA/QC can be found in Supplementary Table S2.

### Statistical analysis

Unless otherwise specified, all tests were performed in R [43]. The impact of land conversion on functional genes associated with microbial nutrient cycling was evaluated using two-way ANOVAs, with time since conversion (forest, < 10 y in agriculture, > 10 and < 50 y in agriculture, and > 50 y in agriculture) and depth intervals (0–5 cm, and 5–15 cm) as fixed factors. Data was then analyzed using one-way ANOVAs followed by Tukey post hoc tests (*p* < 0.05), to evaluate time groups within a depth increment. Data distribution and homoscedasticity was assessed using Shapiro-Wilk and Levene tests. Non-normal data were evaluated using the Kruskal-Wallis test followed by the Wilcoxon test using the p-value adjustment Bonferroni method. The qPCR data was analyzed on a copy number ng^− 1^ DNA basis (Supplementary Table S3); data provided on a per gram of soil basis is available in the Supplementary materials (Supplementary Tables S4 and S5).

To assess the influence of time since conversion and soil depth on overall community structure, Non-Parametric Multidimensional Scaling (NMDS) ordinations based on Bray-Curtis dissimilarities were constructed for each dataset (bacterial and fungal OTUs) using the vegan package in R (v2.6-2). The top 10 most abundant phyla were overlaid onto ordination space to assess overall phyla level shifts based on time since land conversion. Differences in time since conversion was assessed using PERMANOVA (“adonis” function; “vegan” package) on the overall bacterial and fungal community datasets as well as for each depth increment (Supplementary Table S6). Pairwise assessment of differences in groupings was done using the “pairwise.perm.manova” function in the “RVAideMemoire” package in R. P-values were adjusted for multiple comparisons using Benjamini-Hochberg corrections.

Changes in overall community diversity were evaluated using univariate approaches. Fungal and bacterial Shannon and Simpsons diversity indices were generated using QIIME 2 [44] and then evaluated using ANOVA or Kruskal-Wallis tests (normality assessed as indicated above), followed by Tukey HSD or pairwise Wilcoxon *post hoc* tests using the p-value adjustment Bonferroni method. Bacterial 16S rRNA and fungal ITS relative abundances were summarized at the class level and significant shifts were evaluated as outlined above. The relationship between soil microbial taxonomic and functional communities and soil physico-chemical properties (Supplementary Table S7 and [8]) was assessed using Spearman’s rank correlation.

## Results

### Overall change in microbial abundance and functional potential

**Fig. 1:**
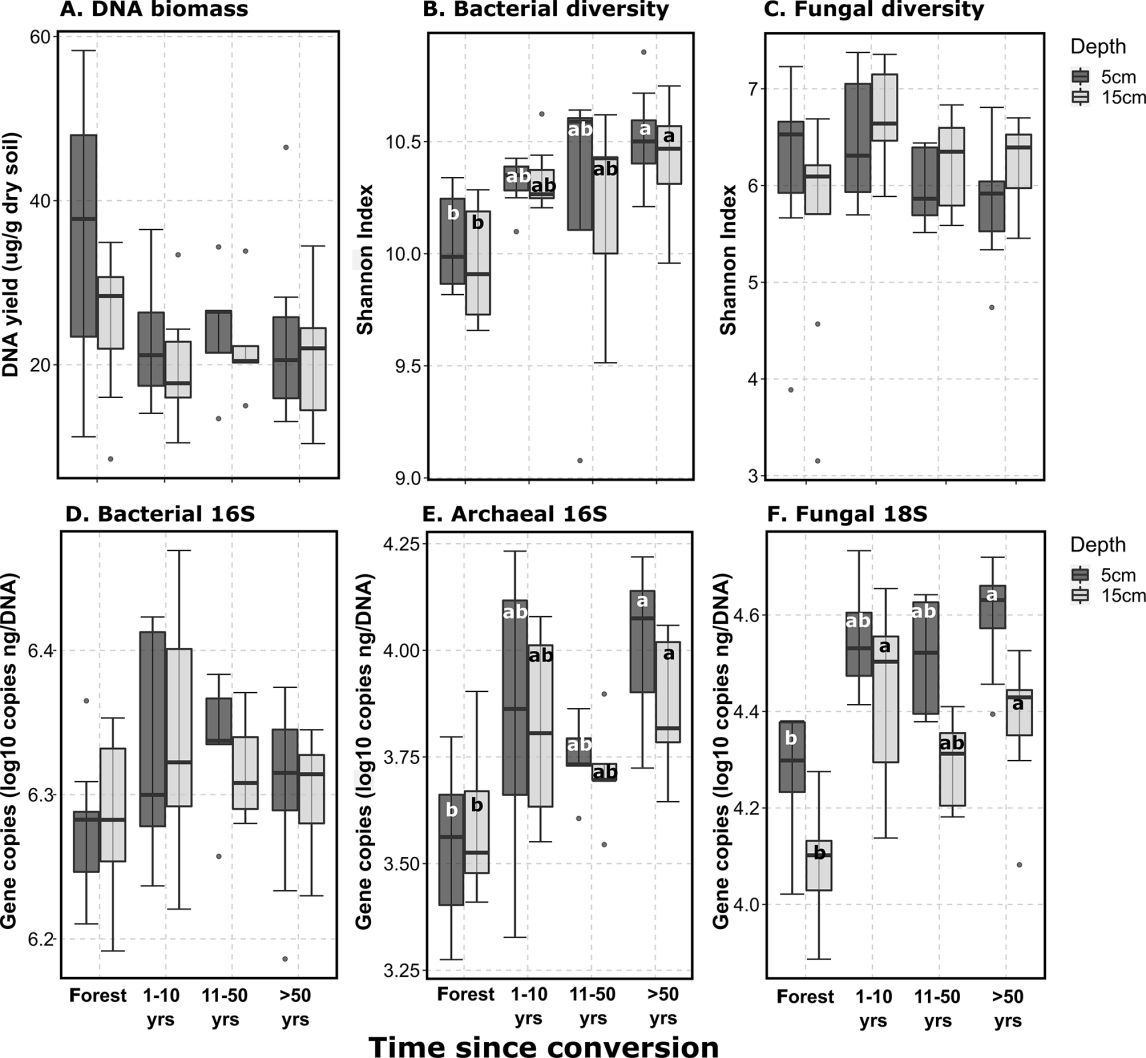
Abundance and diversity of soil microbial communities after forest conversion. **A**: Molecular microbial biomass (DNA yield); **B**-**C**: bacterial and fungal Shannon diversity indices; **D**-**F**: bacterial 16S rRNA, archaeal 16S rRNA, and fungal 18S rRNA gene copies ng^-1^ DNA. Significant differences (*p* < 0.05) within an individual depth increment (5–15 cm) are denoted by different letters. Forest *n* = 9, 1–10 years *n* = 8, 11–50 years *n* = 5, > 50 years *n* = 8

Microbial abundance and function differed both with time since conversion and by soil depth. Microbial molecular biomass generally decreased after land conversion from an average high of 30 µg DNA g^−1^ dry soil in forest soils to an average low of 20 µg DNA g^−1^ dry soil in agricultural soils (Fig. 1A). Bacterial alpha diversity (Fig. 1B) within this microbial biomass increased over time at both soil depths, but fungal alpha diversity (Fig. 1C) was not significantly impacted. The increase in bacterial diversity was not accompanied by an increase in the relative abundance of bacteria 16S rRNA genes, which remained relatively constant at 1.8 × 10^7^ 16S rRNA copies ng^−1^ DNA (Fig. 1D). In contrast, the relative abundance of archaea (*p* < 0.05) and fungi (*p* < 0.001) taxonomic genes increased more than 70% at both soil depths as the time since conversion increased (Fig. 1E, F).

**Fig. 2:**
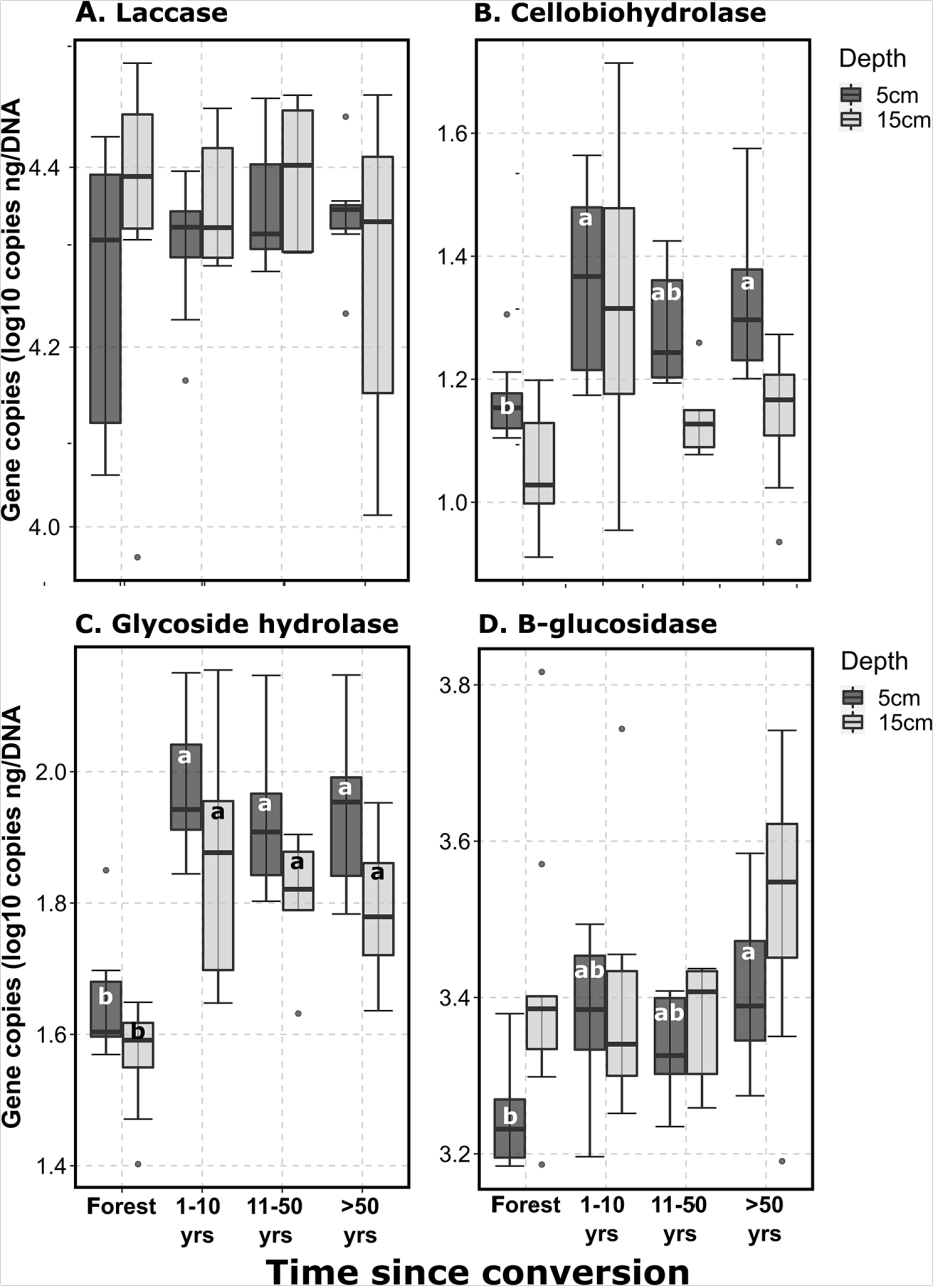
Shifts in bacterial and fungal carbon cycling functions after forest conversion. **A**: laccase (bacteria and fungi); **B**: Cellobiohydrolase (fungi); **C**: Glycoside hydrolase (fungi); **D**: β-glucosidase (bacteria). Quantitative PCR data shown as gene copies ng^-1^ DNA. Significant differences (*p* < 0.05) within an individual depth increment (5–15 cm) are denoted by different letters. Forest *n* = 9, 1–10 years *n* = 8, 11–50 years *n* = 5, > 50 years *n* = 8

The relative abundance of functions associated with complex organic matter decomposition (Fig. 2A; laccase) did not change after conversion, but those associated with lower molecular weight C cycling increased. Genes associated with the breakdown of cellulolytic compounds (cellobiohydrolase; Fig. 2B), hemicellulose and cellulose (glycoside hydrolase; Fig. 2C), and oligosaccharides (β-glucosidase; Fig. 2D) increased on average by 49, 103, and 33%, respectively, in the surface soils (*p* < 0.05); glycoside hydrolase also showed significant increases in the lower soil depths (Fig. 2C; average increase of 83%).

**Fig. 3:**
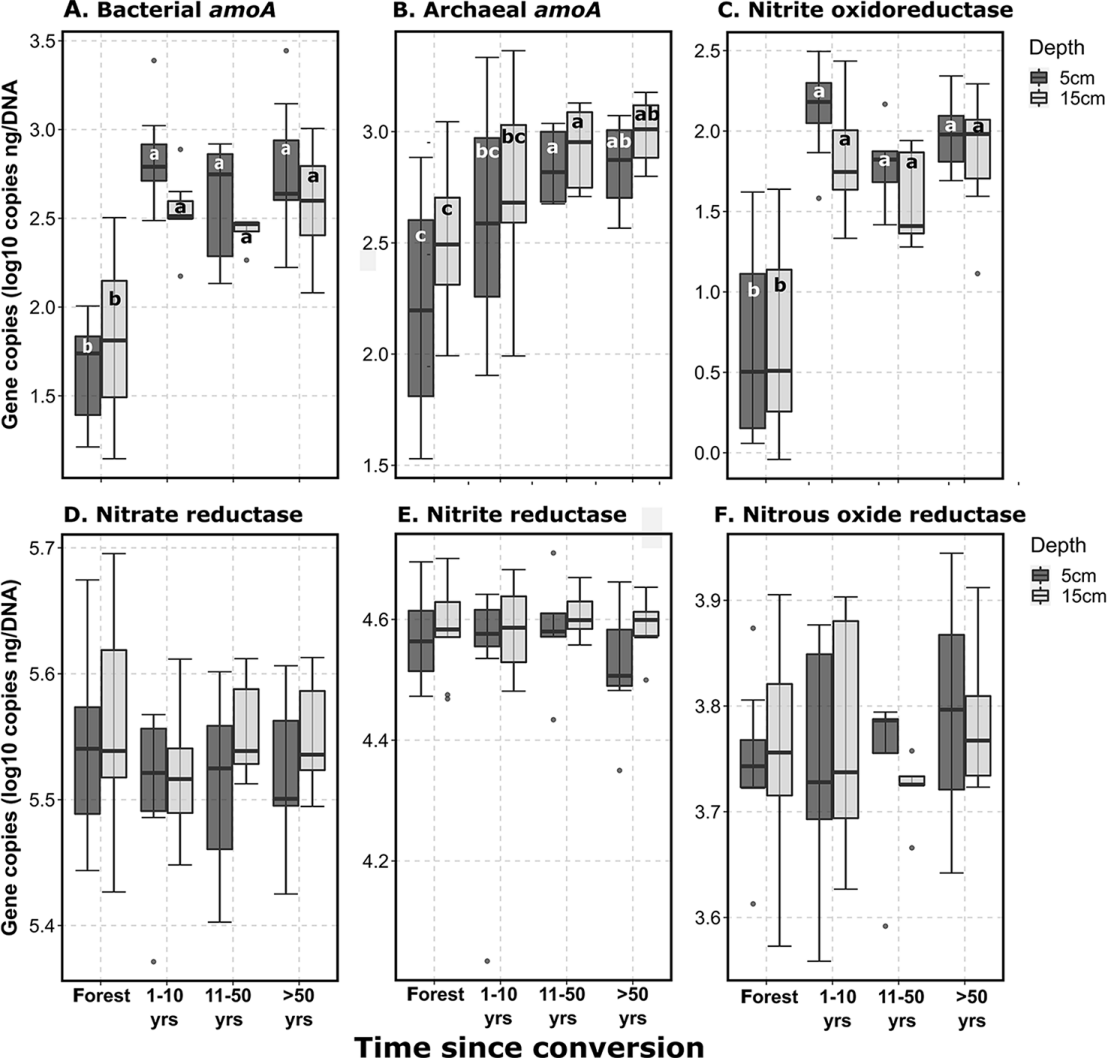
Shifts in bacterial and archaeal nitrogen cycling functions after forest conversion. Quantitative PCR data shown as gene copies ng^-1^ DNA. Significant differences (*p* < 0.05) within an individual depth increment (5–15 cm) are denoted by different letters. *amoA*, ammonium monooxygenase. All assays except Archaeal *amoA* target bacterial functions. Forest *n* = 9, 1–10 years *n* = 8, 11–50 years *n* = 5, > 50 years *n* = 8

Similar to patterns seen with lower molecular weight C functions, bacterial and archaeal functional genes associated with nitrification (N cycling) all significantly increased after land conversion (Fig. 3A-C; *p* < 0.001), with increases ranging from a low of 112% for archaeal ammonia monooxygenase genes (5–15 cm soil depth) to a high of 1310% for bacterial ammonia monooxygenase genes (0–5 cm soil depth). All other assessed N cycling genes, including those associated with ammonification (nitrate reductase; Fig. 3D) and denitrification (nitrite reductase and nitrous oxide reductase; Fig. 3E-F and Supplementary Tables S3) did not significantly change after land conversion.

**Fig. 4:**
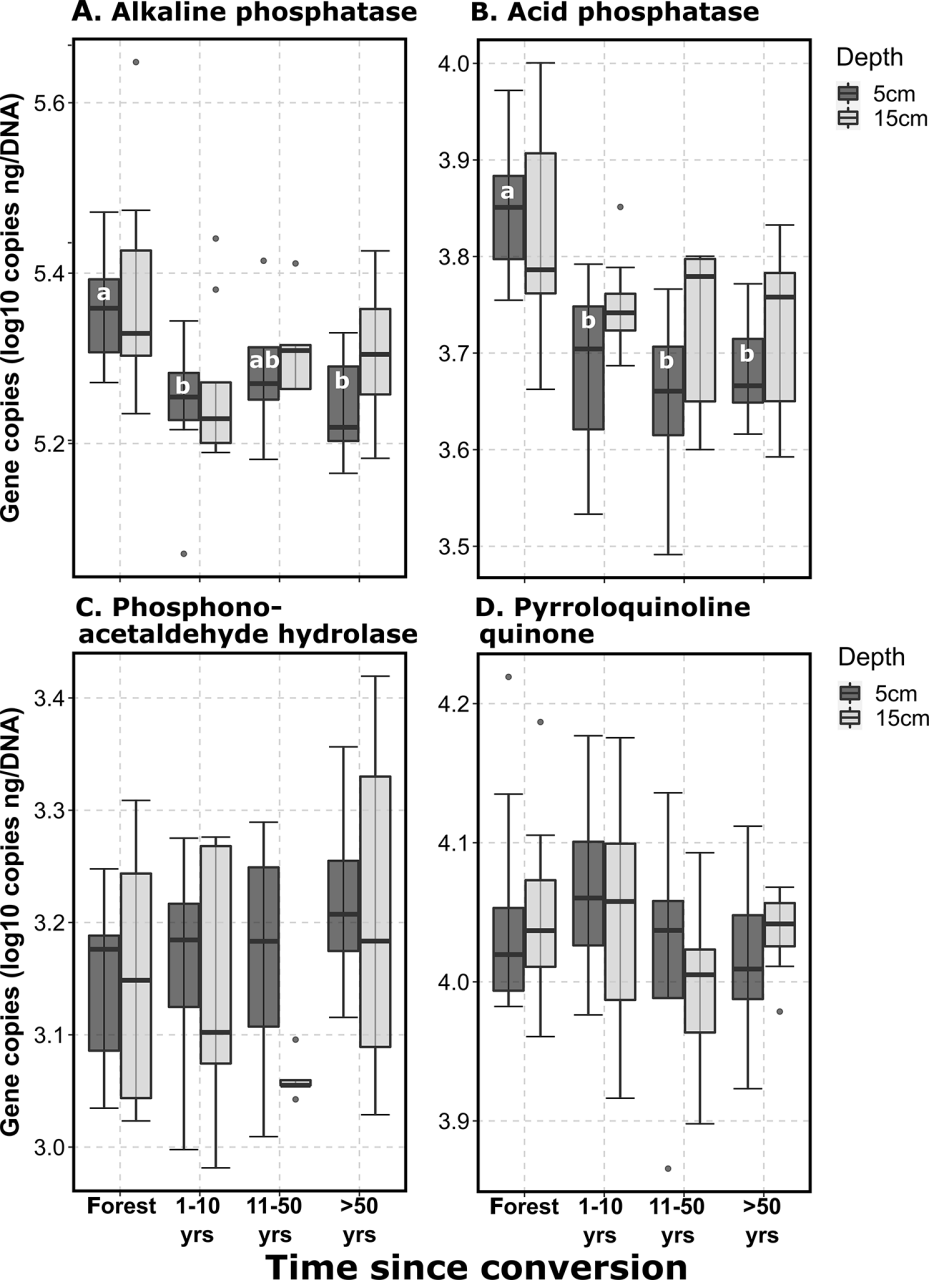
Shifts in bacterial phosphorous cycling functions after forest conversion. Quantitative PCR data shown as gene copies ng^-1^ DNA. Significant differences (*p* < 0.05) within an individual depth increment (5–15 cm) are denoted by different letters. Forest *n* = 9, 1–10 years *n* = 8, 11–50 years *n* = 5, > 50 years *n* = 8

Finally, changes in genes associated with different P cycling functions were variable. The relative abundance of functional genes associated with P mineralization decreased after land conversion (Fig. 4A, B). These changes were significant in the surface soils (*p* < 0.001), with average decreases of 20 and 33%, respectively for alkaline and acid phosphatase. The other two assessed genes associated with P cycling, phosphono-acetaldehyde hydrolase (C-P bond cleavage) and pyrroloquinoline quinone (associated with P solubilization) were not affected by land clearing.

### Shifts in bacterial and fungal communities following land conversion

**Fig. 5:**
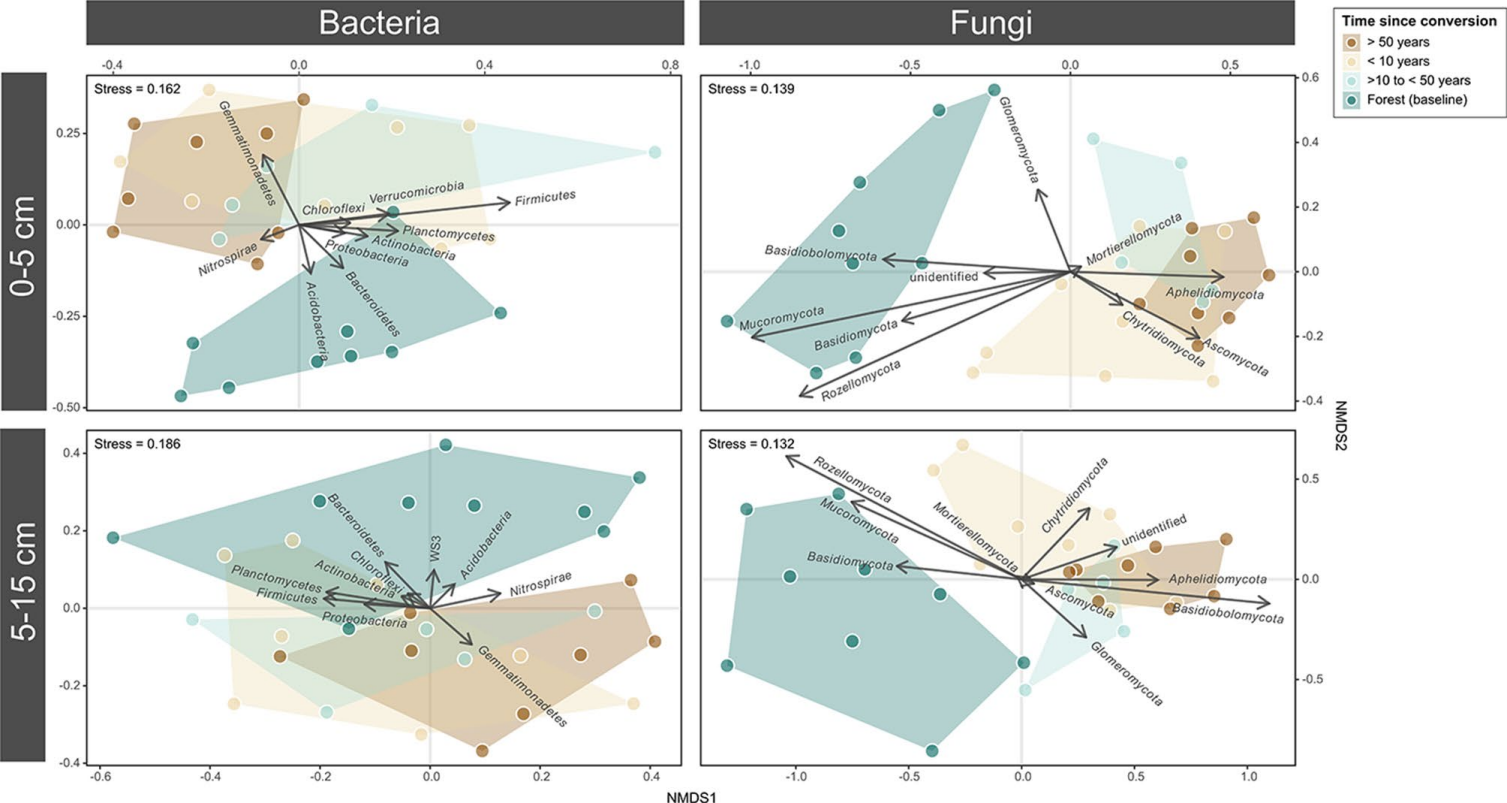
Overall shifts in bacterial and fungal communities after conversion from forest to agriculture. The prevalence of the top ten most abundant phyla for each depth subset are shown as vectors. NMDS ordinations were based on Bray-Curtis dissimilarities

Unique bacterial and fungal communities developed after land conversion (Fig. 5). Bacterial community structure in the reference forested sites was significantly different from the agricultural systems (*p* < 0.042) in both surface and sub-surface soils. When examined across both depths, communities in the forest and > 50 years post-conversion sites each clustered separately than all other systems (*p* < 0.025) while the 1–10 and 11–50 year systems were similar (*p* = 0.116, Supplementary Table S6). The discrete clustering of forest bacterial communities at both depths was associated with increased Acidobacteriota, Actinomycetota, and Bacteroidota OTUs, while those of agriculture systems were associated with increased Gemmatimonadota OTUs (Fig. 5). Similarly, fungal community composition in the forested sites was significantly different from the other systems, both within each depth increment (Fig. 5, *p* < 0.002) and when examined across both depths, where all systems were significantly different (*p* < 0.027, Supplementary Table S6). The distinct clustering of forest fungal communities at both depths was associated with increased Basidiomycota and Mucoromycota OTUs, while those of agriculture systems were associated with increased Ascomycota OTUs (Fig. 5).

**Fig. 6:**
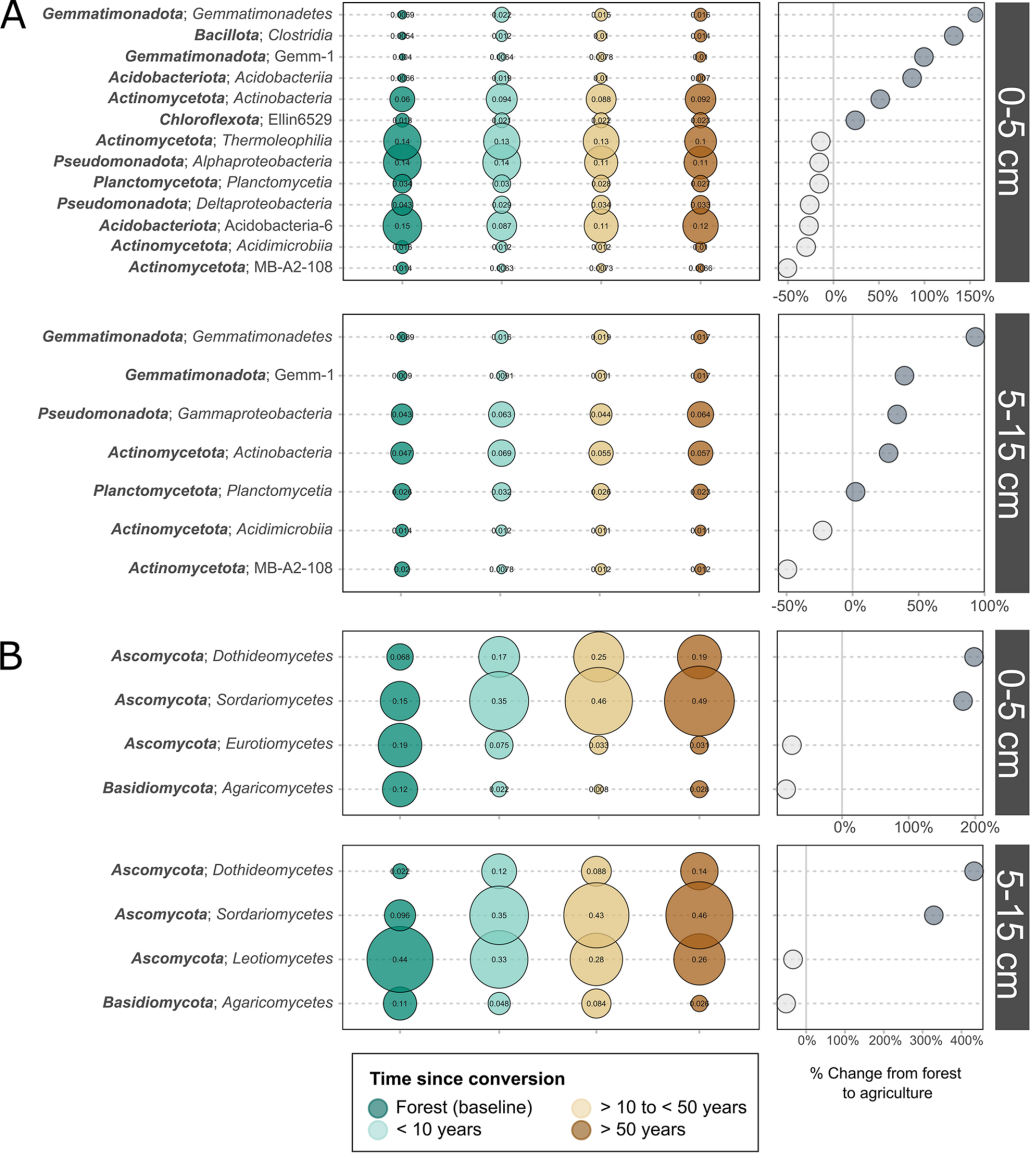
Change in the relative abundance of microbial taxonomic classes after conversion from forest to agriculture. (**A**) Bacteria; (**B**) Fungi. Only class level shifts showing significant differences based on time since conversion (*p* < 0.05; ANOVA) within each depth increment are shown. The magnitude of the change in mean relative abundance from the forested sites to agricultural fields after greater than 50 years is shown in the right panel

Bacterial communities in the surface soils experienced more significant shifts at the class level than those in the sub-surface (Fig. 6A). The relative abundance of the Bacillota class Clostridia more than doubled in the 0–5 cm agricultural soils relative to the forest soils. At both depths two Actinomycetota classes (Acidimicrobiia, MB.A2.108) decreased in relative abundance after conversion to agriculture, while two Gemmatimonadota classes (Gemmatimonadetes, Gemm.1) increased in relative abundance. In fungal communities, the clear shift towards an Ascomycota-dominated system (Fig. 5) was associated with highly significant increases in the relative abundance of Ascomycota classes Sordariomycetes and Dothideomycetes, which increased by 329% and 433% (respectively) in the deeper soils and by 182% and 198% (respectively) in the surface soils (Fig. 6B). The relative abundance of the Basidiomycota class Agaricomycetes decreased after forest conversion by 84% and 52% in the surface and deeper soils, respectively (Fig. 6B).

### Relationship between soil organic matter, soil chemical properties, and soil biology

Spearman’s rank correlation was used to assess the interactions between soil properties (Supplementary Table S7 and Benalcazar et al., 2022) and microbial taxonomic and functional gene abundance (Table 1). The overall functional potential of the soil microbial communities to break down low molecular weight carbon compounds (β-glucosidase) was negatively correlated with SOM and C + N pools, and positively correlated with pH. In contrast, the cellulolytic (cellobiohydrolase) potential decreased with increasing pH and both cellulolytic and hemicellulolytic (glycoside hydrolase) potential increased with increased P and K availability. Functions associated with nitrification (ammonium monooxygenase; nitrite oxidoreductase) were negatively correlated with SOM and total C while those associated with N immobilization (dissimilatory nitrite reductase) and P mineralization (acid phosphatase) were positively correlated with SOM and total C.

**Table 1:** Relationship between soil properties and the relative abundance of microbial taxonomic and functional genes

Function	Gene	SOM	pH	P	K	Total C	Total N	Mg	Fe	Mn	Zn
Taxonomic	Fungal 18 S rRNA	-0.26*		0.65***	0.40**						0.27*
	Archaeal 16 S rRNA			0.47***	0.28*				-0.38**		0.26*
Carbon cycling	Laccase							0.30*			
	Cellobiohydrolase		-0.28*	0.51***	0.27*						
	β-glucosidase	-0.29*	0.56***			-0.32*	-0.35**		-0.44***	-0.50***	
	Glycoside hydrolase	-0.35**		0.56***	0.27*	-0.28*					
Nitrogen cycling	Bacterial ammonia monooxygenase	-0.45***		0.50***		-0.40**	-0.34**		-0.30*		
	Archaeal ammonia monooxygenase	-0.31*	0.37**			-0.38**	-0.35**		-0.40**	-0.61***	
	Nitrite oxidoreductase	-0.46***		0.42***		-0.42***	-0.34**	-0.35**			
	Dissimilatory nitrite reductase	0.29*	0.57***			0.31*		0.49***	-0.60***		
	Nitrous oxide reductase		0.30*	0.31*	0.32*			0.30*	-0.39**		
Phosphorous cycling	Acid phosphatase	0.35**				0.37**	0.26*				
	Pyrroloquinoline quinone	-0.27*									
	Alkaline phosphatase							0.36*			

Relationships assessed using Spearman correlation analysis, assessed across all time and depth intervals. Only genes with significant correlations (*p* < 0.05) to different soil parameters are shown. * *p* < 0.05, ** *p* < 0.01, *** *p* < 0.001. Quantitative PCR information is available in Supplementary Table S1

Within the bacterial communities, two Acidobacteriota classes (Acidimicrobiia, Acidobacteria.6), Actinomycetota class MB.A2.108, and Planctomycetia were positively correlated with SOM, with most also positively correlated with Total C and N (Table 2). A separate Acidobacteriota class, Acidobacteriia, and the Gemmatimonadota class Gemm.1 were negatively correlated with the same parameters. Nitrospira was not correlated with C and N pools, but was negatively associated with Fe, Mn, and Zn. In contrast Planctomycetia and Alphaproteobacteria were positively correlated with these micronutrients. The fungal class Sordariomycetes was negatively associated with SOM and total N, but positively associated with P and K, while class Agaricomycetes showed reverse patterns.

**Table 2:** Relationship between soil properties and the relative abundance of bacterial and fungal classes

Phyla	Class	SOM	pH	P	K	Total C	Total N	Mg	Fe	Mn	Zn
Acidobacteriota	Acidobacteria.6	0.32*	0.57***			0.30*		0.38**	-0.6***		
	Acidobacteriia	-0.40**	-0.50***			-0.40**	-0.30*	-0.30*	0.58***		
	Chloracidobacteria		0.40**						-0.30*	-0.50***	
	Acidimicrobiia	0.58***		-0.30*		0.59***	0.53***			0.31*	
Actinomycetota	Actinobacteria		-0.30*	0.64***	0.49***						0.30*
	MB.A2.108	0.26*	0.37**	-0.40**				0.46***			
Chloroflexi	Ellin6529	-0.30*			-0.40**	-0.30*	-0.30*	-0.30*		-0.30*	0.33*
Gemmatimonadota	Gemm.1	-0.40**	0.45***			-0.40***	-0.40***			-0.50***	
Nitrospirota	Nitrospira								-0.30*	-0.40***	-0.40**
Planctomycetota	Planctomycetia	0.28*	-0.60***			0.30*	0.28*		0.34**	0.40**	0.31*
Pseudomonadota	Alpha-		-0.70***						0.59***	0.36**	0.27*
	Delta-			-0.40**							
	Gamma-			0.31*				-0.50***			
Ascomycota	Dothideomycetes			0.33*							
	Leotiomycetes			-0.50***	-0.40**				0.36**		
	Sordariomycetes	-0.30*		0.49***	0.31*		-0.30*		-0.30**	-0.40**	
Basidiomycota	Agaricomycetes	0.32*		-0.40**		0.31*			0.26*	0.34**	

Relationships assessed using Spearman correlation analysis, assessed across all time and depth intervals. Only taxa with significant correlations (*p* < 0.05) to different soil parameters are shown for each bacterial and fungal class * *p* < 0.05, ** *p* < 0.01, *** *p* < 0.001

## Discussion

Land conversion to agriculture at the southern limit of the boreal region (Thunder Bay, Ontario) altered both the abundance and functionality of the soil microbial communities, with the magnitude and direction of change differing with time and with soil depth. Molecular microbial biomass, or the amount of extractable DNA, generally decreased by 31% in surface soils and 18% in subsurface soils, mirroring previously reported patterns of organic matter loss in this system [8]. Land conversion to agriculture across the globe has been shown to alter microbial biomass [16] as the clearing of vegetation, site preparation, and subsequent tillage typically depletes organic matter and increases compaction in surface soils [5, 6].

The general decrease in microbial biomass after conversion was not accompanied by comparable decreases in either microbial diversity (assessed by sequencing) or relative abundance (assessed by quantitative PCR). Fungal diversity was maintained and the relative abundance of fungi and archaea within the microbial biomass increased, while bacterial diversity increased and the relative abundance of bacteria was maintained. A recent study on converted systems in the Yukon Territory similarly found that archaeal abundance increased, and bacterial abundance did not change [10]. In contrast to our findings however, Schroeder et al. [10] found that fungal abundance in both pasture- and crop-based agricultural systems was reduced relative to the adjacent forest. However, substantive difference in climate, agricultural practices, and soil type between the two studies, including the presence of permafrost in most of the forested Yukon sites and horticultural cropping practices in the Yukon cropland sites [9, 10], makes direct comparisons challenging. Management practices in the converted Ontario agricultural systems included the use of manure and mineral fertilizers, crop rotation (commonly with alfalfa, corn, barley, and winter wheat), and conservation tillage [8]. These moderate intensity management practices, which alter the soil structure and nutrient balances of the initial system, have been shown to increase bacterial and fungal abundance [22] and diversity [11, 13, 45] in agricultural soils, in part because they generate increased niche and resource heterogeneity.

The overall microbial community structure was significantly different in the agricultural systems, and these shifts occurred rapidly after conversion. Fungal communities were rapidly altered, but significantly different communities were found in each of the post-conversion age increments as Ascomycota-Dothideomycetes and -Sordariomycetes continued to increase and Ascomycota-Eurotiomycetes and -Leotiomycetes continued to decrease in relative abundance. Basidiomycota, and in particular the class Agaricomycetes which includes many conifer-associated ectomycorrhizal fungi [46], also significantly decreased as the system shifted from forest dominant to crop dominant. These substantive shifts in fungal groups have implications both for carbon cycling as well as for agricultural soil health. For example, both Sordariomycetes and Dothideomycetes are diverse classes of fungal decomposers, like most Ascomycota, but both also include important crop pathogens such as *Fusarium* and *Phaeosphaeria* spp [47], which are known to generally increase in Ontario grain cropping systems [42].

Bacterial community structure in the surface soils was also altered within the first few years after land conversion, followed by several decades of transition before stabilizing more than 50 years post-conversion. At the phyla level, the relative abundance of Acidobacteriota, Actinomycetota, Bacteroidota, and Pseudomonadota decreased, while the abundance of Gemmatimonadota increased. Previous studies have shown that Acidobacteriota, Actinomycetota, Pseudomonadota are dominant phyla in boreal soils [27], and that increased pH due to the addition of wood ash and liming in agricultural fields reduces their abundance [48]. Within each phylum however, different shifts in the relative abundance of classes occurred. For example, Actinomycetota-Actinobacteria increased by up to 50% in surface and subsurface soils, while Actinomycetota-MB-A2-108 decreased by approximately the same amount. There was a significant relationship between pH and many of the bacterial classes that either increased (positive association) or decreased (negative association) post-conversion. Altered pH, which in general co-occurred with altered OM levels as found in other boreal systems [15], is well known to be a dominant factor influencing the composition of bacterial communities [14], but within a given system multiple complex factors come into play. For example, MB-A2-108 was positively associated with pH yet still decreased in converted systems, and its overall abundance was more strongly controlled by soil fertility (e.g., P levels), as has also been found in other agricultural systems [49]. The greatest shift in relative abundance occurred in Gemmatimonadota. This phylum has been shown to be more abundant in agricultural soils globally [50], with an increased abundance believed to be promoted due to generally higher nutrient status and more neutral pH, relative to forest soils. Since different Gemmatimonadota are known to have the genetic potential to reduce N_2_O to N_2_ [50], their increase in northern agricultural soils could act to mitigate N-based GHG emissions as agriculture in these regions expands.

Although changes in overall bacterial and fungal communities were highly significant post-conversion, their functional complexity means that these taxonomic level changes may not be useful to evaluate how forest conversion will alter C and N cycling dynamics, as most bacterial and fungal taxa are involved in C and N cycling at some level [18, 27, 51, 52]. Instead, co-associated shifts in different biogeochemical cycling functional potential may serve as better indicators of overall soil health change.

Land conversion had differing effects on the capacity of the soil communities to cycle different carbon compounds. Bölscher et al. [53] previously reported that microbial communities in forest soils were more efficient in using lower molecular weight (LMW) C substrates, but Zhou et al. [16] found that C-decomposition increased following forest disturbance. In the northern Ontario we found that the relative abundance of genes associated with complex OM decomposition (laccase) did not change, but the relative abundance of genes associated with the decomposition of LMW-C compounds increased with the switch to high inputs of bioavailable crop C (roots and crop residues) and nutrients (e.g. N and P). Functional shifts in fungal genes associated with C cycling stabilized within 10 years while bacterial genes associated with C cycling (B-glucosidase) were still in undergoing shifts decades later. Although we did not directly measure how different C-pools changed after land conversion, compounds like cellulose, hemicellulose, and sugars are common in agricultural fields [54, 55], where C is more abundant as bio-available particulate organic matter relative to more stable mineral-associated organic matter [5]. Schroeder et al. [10] found that microbial carbon use efficiency (CUE) increased following forest conversion but suggested that increased CUE was primarily mediated through abiotic factors rather than altered microbial communities, as they found no difference in the relative abundance of bacteria or fungi post-conversion. In our study, fungal abundance clearly increased post-conversion, and the significant shifts in the capacity of both bacteria and fungi to cycle LMW-C would contribute to increased CUE.

Functions associated with N and P cycling also shifted after land conversion, as both mineral and organic fertilizer inputs altered soil nutrient status [8]. The relative abundance of genes associated with nitrification (*amoA* and *nxrA*) generally increased, however genes associated with denitrification were not impacted by forest to agricultural conversion. Soil management practices including tillage, organic amendments, and fertilizer N are well known to increase the abundance of both bacterial and archaeal nitrifiers [57, 58]. Although archaeal ammonia oxidizers dominate nitrification when ammonia is produced by the mineralization of organic N rather than fertilizer N, these nitrifiers have also been shown to increase in response to N application in acidic soils [59]. In addition, nitrifier abundance increases in soils amended with cattle manure [60], which is a common practice among dairy farmers in the study region [8]. Functions associated with organic P mineralization potential (acid and alkaline phosphatase) followed opposite trends to those seen with nitrification functions and decreased after conversion. Increased stores of extractable P, which occur when converted forest soils are fertilized [6, 8], have been shown to lead to reduced alkaline phosphatase abundance [61]. Although there was no direct relationship between phosphatase gene abundance and extractable P at our site, acid phosphatase abundance was positively associated with soil organic matter. This finding suggests that a shift from mainly organic P to mineral P contributed to the observed functional changes, as has been seen in other systems [62, 63].

## Conclusions

In this study, we found that forest conversion to agriculture in boreal soils affected microbial abundance, diversity, composition, and functional potential. Although substantive changes began to occur soon after land conversion to agriculture, microbial community structure and functional potential continued to change for decades. Bacterial nitrification processes increased within the first decade after which they stabilized, but C cycling processes were still changing 50 years post conversion. In general however, variability in both community structure and functional potential decreased after the first decade and new stable states [28] began to emerge. The type and timeline of changes are similar to those that occur when land use change occurs in the other direction, for example when previously cropped soils are converted to forests. In afforested systems, it similarly takes decades for microbial abundance and C cycling processes to stabilize [64–66]. Our findings highlight how C, N, and P cycling processes may be altered as agriculture expands northward into forested regions of Canada and provides insight into how better to manage these systems to ensure their sustainability.

### Electronic supplementary material

Below is the link to the electronic supplementary material.

Supplementary Material 1

## Data Availability

The sequence data supporting the conclusions of this article are available in NCBI Short Read Archive under Project number PRJNA1062837. The chemical and quantitative PCR datasets supporting the conclusions of this article are included within the article and its additional files.

## References

[1] Bush E, Lemmen DS (2019). Canada’s changing climate report.

[2] King M, Altdorff D, Li P, Glagedara L, Holden J, Unc A (2018). Northward shift of the agricultural climate zone under 21st-century global climate change. Sci Rep.

[3] Unc A, Altdorff D, Abakumov E, Adl S, Baldursson S, Bechtold M (2021). Expansion of agriculture in northern cold-climate regions: a cross-sectoral perspective on opportunities and challenges. Front Sustain Food Syst.

[4] Altdorff D, Borchard N, Young EH, Galagedara L, Sorvali J, Quideau S (2021). Agriculture in boreal and Arctic regions requires an integrated global approach for research and policy. Agron Sustain Dev.

[5] DeGryze S, Six J, Paustian K, Morris SJ, Paul EA, Merckx R (2004). Soil organic carbon pool changes following land-use conversions. Glob Change Biol.

[6] Ellert B, Gregorich EG (1996). Storage of carbon, nitrogen and phosphorus in cultivated and adjacent forest soils of Ontario. Soil Sci.

[7] Reicosky D. Managing soil health for sustainable agriculture volume 1: fundamentals. 1st ed. Burleigh Dodds Science Publishing; 2018.

[8] Benalcazar P, Diochon A, Kolka R, Schindelbeck R, Sahota T, McLaren BE (2022). The impact of land conversion from boreal forest to agriculture on soil health indicators. Can J Soil Sci.

[9] Peplau T, Schroeder J, Gregorich E, Poeplau C (2022). Subarctic soil carbon losses after deforestation for agriculture depend on permafrost abundance. Glob Change Biol.

[10] Schroeder J, Peplau T, Pennekamp F, Gregorich E, Tebbe CC, Poeplau C (2024). Deforestation for agriculture increases microbial carbon use efficiency in subarctic soils. Biol Fertil Soils.

[11] Tardy V, Spor A, Mathieu O, Lévèque J, Terrat S, Plassart P (2015). Shifts in microbial diversity through land use intensity as drivers of carbon mineralization in soil. Soil Biol Biochem.

[12] Mäkipää R, Abramoff R, Adamczyk B, Baldy V, Biryol C, Bosela M (2023). How does management affect soil C sequestration and greenhouse gas fluxes in boreal and temperate forests? – a review. Ecol Manag.

[13] de Graaff M-A, Hornslein N, Throop HL, Kardol P, van Diepen LTA (2019). Effects of agricultural intensification on soil biodiversity and implications for ecosystem functioning: a meta-analysis. Adv Agron.

[14] Fierer N (2017). Embracing the unknown: disentangling the complexities of the soil microbiome. Nat Rev Microbiol.

[15] Giguère-Tremblay R, Laperriere G, de Grandpré A, Morneault A, Bisson D, Chagnon P-L (2020). Boreal forest multifunctionality is promoted by low soil organic matter content and high regional bacterial biodiversity in Northeastern Canada. Forests.

[16] Zhou Z, Wang C, Luo Y (2018). Effects of forest degradation on microbial communities and soil carbon cycling: a global meta-analysis. Glob Ecol Biogeogr.

[17] Bauhus J, Pare D, Côté L (1998). Effects of tree species, stand age and soil type on soil microbial biomass and its activity in a southern boreal forest. Soil Biol Biochem.

[18] Merloti LF, Mendes LW, Pedrinho A, de Souza LF, Ferrari BM, Tsai SM (2019). Forest-to-agriculture conversion in Amazon drives soil microbial communities and N-cycle. Soil Biol Biochem.

[19] Pankhurst CE, Doube BM, Pankhurst CE, Doube BM, Gupta VVSR (1997). Biological indicators of soil health: synthesis. Biological indicators of soil health.

[20] Bevivino A, Paganin P, Bacci G, Florio A, Pellicer MS, Papaleo MC (2014). Soil bacterial community response to differences in agricultural management along with seasonal changes in a Mediterranean region. PLoS ONE.

[21] Peltoniemi K, Velmala S, Fritze H, Lemola R, Pennanen T (2021). Long-term impacts of organic and conventional farming on the soil microbiome in boreal arable soil. Eur J Soil Biol.

[22] Morugán-Coronado A, Pérez-Rodríguez P, Insolia E, Soto-Gómez D, Fernández-Calviño D, Zornoza R (2022). The impact of crop diversification, tillage and fertilization type on soil total microbial, fungal and bacterial abundance: a worldwide meta-analysis of agricultural sites. Agric Ecosyst Environ.

[23] Hicks LC, Rousk K, Rinnan R, Rousk J (2020). Soil microbial responses to 28 years of nutrient fertilization in a subarctic heath. Ecosystems.

[24] Neurauter M, Yuan M, Hicks LC, Rousk J (2023). Soil microbial resource limitation along a subarctic ecotone from birch forest to tundra heath. Soil Biol Biochem.

[25] Allison SD, Czimczik CI, Treseder KK (2008). Microbial activity and soil respiration under nitrogen addition in alaskan boreal forest. Glob Change Biol.

[26] Kibblewhite MG, Ritz K, Swift MJ (2008). Soil health in agricultural systems. Philos Trans R Soc.

[27] Lladó S, López-Mondéjar R, Baldrian P (2017). Forest soil bacteria: diversity, involvement in ecosystem processes, and response to global change. Microbiol Mol Biol Rev.

[28] Shade A, Peter H, Allison S, Baho D, Berga M, Buergmann H (2012). Fundamentals of microbial community resistance and resilience. Front Microbiol.

[29] Burnett MS, Schütte UME, Harms TK (2022). Widespread capacity for denitrification across a boreal forest landscape. Biogeochemistry.

[30] Seitz TJ, Schütte UME, Drown DM (2022). Unearthing shifts in microbial communities across a disturbance gradient. Front Microbiol.

[31] Truu M, Nõlvak H, Ostonen I, Oopkaup K, Maddison M, Ligi T (2020). Soil bacterial and archaeal communities and their potential to perform n-cycling processes in soils of boreal forests growing on well-drained peat. Front Microbiol.

[32] Paavolainen L, Smolander A (1998). Nitrification and denitrification in soil from a clear-cut Norway spruce (*Picea abies*) stand. Soil Biol Biochem.

[33] Paavolainen L, Kitunen V, Smolander A (1998). Inhibition of nitrification in forest soil by monoterpenes. Plant Soil.

[34] Li J, Delgado-Baquerizo M, Wang J-T, Hu H-W, Cai Z-J, Zhu Y-N (2019). Fungal richness contributes to multifunctionality in boreal forest soil. Soil Biol Biochem.

[35] Mollard DG, Mollard JD. Thunder Bay Area (NTS 52A/SW), District of Thunder Bay. In: Northern Ontario Engineering Geology Terrain Study 71. Ontario Geological Survey. 1983. http://www.geologyontario.mndmf.gov.on.ca/mndmfiles/pub/data/imaging/NOEGTS071TS071.pdf. Accessed 10 Nov 2023.

[36] Wester MC, Henson BL, Crins WJ, Uhlig PWC, Gray PA. The ecosystems of Ontario, Part 2: Ecodistricts. Ontario Ministry of Natural Resources and Forestry, Science and Research Branch, Peterborough, ON. Science and Research Technical Report- TR-26. 2018.

[37] Edge TA, Baird DJ, Bilodeau G, Gagné N, Greer C, Konkin D, et al. The Ecobiomics project: advancing metagenomics assessment of soil health and freshwater quality in Canada. Sci Total Environ. 2020;710:135906. https://www.ontario.ca/page/ecosystems-ontario-part-2-ecodistricts. Accessed 10 Nov 2023. 10.1016/j.scitotenv.2019.135906 31926407

[38] Parada AE, Needham DM, Fuhrman JA. Every base matters: assessing small subunit rRNA primers for marine microbiomes with mock communities, time series and global field samples. Environ Microbiol. 2016;18:1403–14. 10.1111/1462-2920.13023 26271760

[39] Quince C, Lanzen A, Davenport RJ, Turnbaugh PJ (2011). Removing noise from pyrosequenced amplicons. BMC Bioinform.

[40] Ihrmark K, Bödeker ITM, Cruz-Martinez K, Friberg H, Kubartova A, Schenck J (2012). New primers to amplify the fungal ITS2 region–evaluation by 454-sequencing of artificial and natural communities. FEMS Microbiol Ecol.

[41] White TJ, Bruns TD, Lee SB, Taylor J, Innis MA, Gelfand DH, Sninsky JJ, White TJ (1990). Amplification and direct sequencing of fungal ribosomal RNA genes for phylogenetics. PCR protocols: a guide to methods and applications.

[42] Pérez-Guzmán L, Phillips LA, Seuradge BJ, Agomoh I, Drury CF, Acosta‐Martínez V (2021). An evaluation of biological soil health indicators in four long‐term continuous agroecosystems in Canada. Agric Ecosyst Environ.

[43] R Core Team. R: A language and environment for statistical computing R. Foundation for Statistical Computing, Vienna, Austria. 2020. https://www.r-project.org/.

[44] Bolyen E, Rideout JR, Dillon MR, Bokulich NA, Abnet CC, Al-Ghalith GA (2019). Reproducible, interactive, scalable and extensible microbiome data science using QIIME 2. Nat Biotechnol.

[45] Trivedi P, Delgado-Baquerizo M, Anderson IC, Singh BK (2016). Response of soil properties and microbial communities to agriculture: implications for primary productivity and soil health indicators. Front Plant Sci.

[46] McGuire KL, Allison SD, Fierer N, Treseder KK (2013). Ectomycorrhizal-dominated boreal and tropical forests have distinct fungal communities, but analogous spatial patterns across soil horizons. PLoS ONE.

[47] Termorshuizen AJ. Ecology of fungal plant pathogens. In: Heitman J, Howlet BJ, Crous PW, Stukenbrock EH, James TY, Gow NAR, editors. The fungal kingdom. ASM; 2017. pp. 387–97.

[48] Reid C, Watmough SA (2014). Evaluating the effects of liming and wood-ash treatment on forest ecosystems through systematic meta-analysis. Can J Res.

[49] Megyes M, Borsodi AK, Árendás T, Márialigeti K (2021). Variations in the diversity of soil bacterial and archaeal communities in response to different long-term fertilization regimes in maize fields. Appl Soil Ecol.

[50] Mujakić I, Piwosz K, Koblížek M (2022). Phylum Gemmatimonadota and its role in the environment. Microorganisms.

[51] López-Mondéjar R, Zühlke D, Becher D, Riedel K, Baldrian P (2016). Cellulose and hemicellulose decomposition by forest soil bacteria proceeds by the action of structurally variable enzymatic systems. Sci Rep.

[52] van Insberghe D, Maas KR, Cardenas E, Strachan CR, Hallam SJ, Mohn WW (2015). Non-symbiotic Bradyrhizobium ecotypes dominate north American forest soils. ISME J.

[53] Bölscher T, Wadsö L, Börjesson G, Herrmann AM (2016). Differences in substrate use efficiency: impacts of microbial community composition, land use management, and substrate complexity. Biol Fertil Soils.

[54] Cherubini F, Ulgiati S (2010). Crop residues as raw materials for biorefinery systems–A LCA case study. Appl Energy.

[55] Ginni G, Kavitha S, Kannah Y, Bhatia SK, Kumar A, Rajkumar M (2021). Valorization of agricultural residues: different biorefinery routes. J Environ Chem Eng.

[56] Attard E, Poly F, Commeaux C, Laurent F, Terada A, Smets BF (2010). Shifts between Nitrospira- and Nitrobacter-like nitrite oxidizers underlie the response of soil potential nitrite oxidation to changes in tillage practices. Environ Microbiol.

[57] Banning NC, Maccarone LD, Fisk LM, Murphy DV (2015). Ammonia-oxidising bacteria not archaea dominate nitrification activity in semi-arid agricultural soil. Sci Rep.

[58] Liang F, Wen Y, Dong X, Wang Y, Pan G, Jiang F (2021). Response of activity and community composition of nitrite-oxidizing bacteria to partial substitution of chemical fertilizer by organic fertilizer. Environ Sci Pollut Res.

[59] Gubry-Rangin C, Kratsch C, Williams TA, McHardy AC, Embley TM, Prosser JI (2015). Coupling of diversification and pH adaptation during the evolution of terrestrial Thaumarchaeota. PNAS.

[60] Tatti E, Goyer C, Chantigny M, Wertz S, Zebarth BJ, Burton DL, Filion M (2014). Influences of over winter conditions on denitrification and nitrous oxide-producing microorganism abundance and structure in an agricultural soil amended with different nitrogen sources. Agric Ecosyst Environ.

[61] Fraser TD, Lynch DH, Gaiero J, Khosla K, Dunfield KE (2017). Quantification of bacterial non-specific acid (*phoC*) and alkaline (*phoD*) phosphatase genes in bulk and rhizosphere soil from organically managed soybean fields. Appl Soil Ecol.

[62] Lang M, Zou W, Chen X, Zou C, Zhang W, Deng Y (2021). Soil microbial composition and *phoD* gene abundance are sensitive to phosphorus level in a long-term wheat-maize crop system. Front Microbiol.

[63] Ragot SA, Kertesz MA, Bünemann EK (2015). PhoD alkaline phosphatase gene diversity in soil. Appl Environ Microbiol.

[64] Luo X, Hou E, Zhang L, Kuan Y, Wen D (2023). Altered soil microbial properties and functions after afforestation increase soil carbon and nitrogen but not phosphorus accumulation. Biol Fertil Soils.

[65] Zhang Q, Wu J, Yang F, Zhang Q, Cheng X (2016). Alteration in soil microbial communities’ composition and biomass following agricultural land use change. Sci Rep.

[66] Zhao FZ, Ren CJ, Han XH, Yang GH, Wang J, Doughty R (2019). Trends in soil microbial communities in afforestation ecosystem modulated by aggradation phases. Ecol Manag.

