# Supplementary material for: Conversion of boreal forests to agricultural systems: soil microbial responses along a land-conversion chronosequence
Source: Environ Microbiome. 2024 May 11;19:32. doi: 10.1186/s40793-024-00576-3 (PMC11088160; doi:10.1186/s40793-024-00576-3)
Supplement: Supplementary file 1 — Supplementary Material 1 [file 40793_2024_576_MOESM1_ESM.docx]

# Supplementary Tables and Information. Environmental Microbiome 2024: Conversion of boreal forests to agricultural systems: Soil microbial responses along a land-conversion chronosequence

**Authors**: Paul Benalcazar^1^, Brent Seuradge^2^, Amanda C. Diochon^3^, Randall K. Kolka^4^, Lori A. Phillips^2*^

^1^ Faculty of Natural Resources Management, Lakehead University, Thunder Bay, ON, Canada

^2^Agriculture and Agri-Food Canada, Harrow Research and Development Centre, ON, Canada

^3^ Department of Geology, Lakehead University, Thunder Bay, ON, Canada

^4^ USDA Forest Services Northern Research Station, Grand Rapid, MN 55744, USA


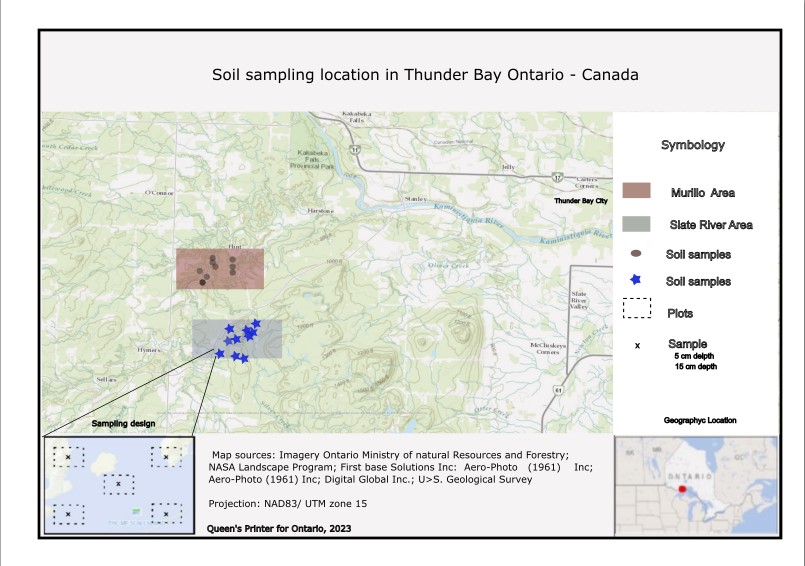


Supplementary Figure S1. Site locations within the Murillo and Slate River regions. At each indicated site, soil was collected from 5 random sub-locations within the field or forested region. Three cores were collected from each of the 5 sub-locations, the 15 cores per site were separated into 0-5 cm and 5-15 cm depth increments, and then each depth increment was composited to form a single surface and sub-surface sample per site (30 sites in total; 60 composite soil samples in total).

SUPPLEMENTARY METHODS: QUANTITATIVE PCR ANALYSES

Table S1. qPCR assays: target organisms, amplification conditions and primer sources, and standard calibration identity.

| **Function^a^** | **Target gene/qPCR assay name** | **Taxonomic Range** | **Primers** | Primer concentration; QPCR reagents | **Cycling conditions^b^** | Efficiency (average) | Best identity of calibration standard source^c^ | **Reference** |
| --- | --- | --- | --- | --- | --- | --- | --- | --- |
| Taxonomic classification: Bacteria | Bacterial 16S | General Bacteria | 16S_341F  /534R | 0.5 μM; Bio-Rad Sso | 35 cycles of 95 °C/5 s, 60 °C/20 s | 96% | *Ralstonia solanacearum*; CP011998.1 | Watanabe et al., 2001 |
| Taxonomic classification:  Fungi | Fungal 18S | General Fungi | Fungal18S-FR1F  /FF390-R | 0.7 μM; BioLine SensiFAST | 40 cycles of 95 °C/5 s, 60 °C/30 s | 91% | *Streptomyces rapamycinicus*; CP006567.1 | Vainio & Hantula, 2000 |
| Taxonomic classification:  Archaea | Archaeal 16S | General Archaea | ARC344F_16S  /Arch806R_16S | 0.5 μM; Bio-Rad Sso | 35 cycles of 95 °C/5 s, 60 °C/20 s | 90% | *Thaumarchaeota archaeon MY3*; CP012850.1 | Raskin et al., 2014 |
| Carbon cycling  (Polyphenolic C breakdown) | Laccase (multicopper oxidase -LMCO) | Bacteria and Fungi | Cu1A-F  /Cu2-R | 1.5 μM; Bio-Rad Sso | 40 cycles of 95 °C/5 s, 60 °C/30 s | 92% | *Stenotrophomonas rhizophila*; CP007597.1 | Kellner et al., 2007 |
| Carbon cycling  (Breakdown of oligosaccharides) | Bglu (β-glucosidase) | Bacteria; wide taxonomic range | Bact-bglu2F  /bglu4R | 0.75 μM; Bio-Rad Sso | 40 cycles of 95 °C/5 s, 60 °C/30 s | 90% | *Limnochorda pilosa*; AP014924.1 | Cañizares et al., 2011 |
| Carbon cycling  (Cellulose decomposition) | cbhI (cellobiohydrolase) | Fungi: Basidiomycota, Ascomycota | fungi-cbhI-F/R | 1.0 μM; BioLine SensiFAST | 40 cycles of 95 °C/5 s, 60 °C/30 s | 86% | *Xylaria polymorpha*; EU345462.1 | Edwards et al., 2008 |
| C+N cycling  (Hemicellulose decomposition) | GH11 (glycoside hydrolase) | Fungi: Basidiomycota, Ascomycota | fungGH11-F/R | 1.0 μM; Bio-Rad Itaq | 40 cycles of 95 °C/5 s, 60 °C/30 s | 100% | *Hericium erinaceu*; HG799590.1 | Barbi et al., 2014 |
| C+N cycling  (Nitrogen mineralization) | apr (alkaline metallopeptidase) | Primarily bacterial | Apr-F/R | 1.2 μM; Bio-Rad Sso | 40 cycles of 95 °C/5 s, 60 °C/30 s | 102% | *Pseudomonas aeruginosa*; CP008873.1 | Bach et al., 2001 |
| Nitrogen cycling  (Nitrification ( NH4+ to NO2-) | *B-amoA*: ammonia monooxygenase | Pseudomonadota | B-amoA-1F/R | 0.5 μM; BioLine SensiFAST | 35 cycles of 95 °C/5 s, 60 °C/20 s | 95% | *Nitrosospira* sp. 9SS1; DQ228455.1 | Rotthauwe et al., 1997 |
| Nitrogen cycling  (Nitrification ( NH4+ to NO2-) | *A-amoA*: ammonia monooxygenase | Archaea | Gen-AOA-F/R | 0.5 μM; Bio-Rad Sso | 35 cycles of 95 °C/5 s, 60 °C/20 s | 97% | *Candidatus* *Nitrosocosmicus*; CP017922.1 | Meinhardt et al., 2015 |
| Nitrogen cycling  (Nitrification: nitrite to nitrate) | nxrA (nitrite oxidoreductase) | Bacteria: Nitrobacter | norA-1F/1R | 0.7 μM; Bio-Rad Sso | 45 cycles of 95 °C/5 s, 60 °C/30 s | 94% | *Nitrobacter winogradskyi*; CP000115.1 | Poly et al., 2008 |
| Nitrogen cycling (Ammonification/Denitrification) | narG: membrane bound nitrate reductase | Bacteria: Pseudomonadota | narG-F/R | 1.0 μM; Bio-Rad Sso | 35 cycles of 95 °C/5 s, 60 °C/30 s | 94% | *Variovorax paradoxus*; CP003912.1 | Gregory et al., 2000 |
| Nitrogen cycling (Dissimilatory nitrate reduction (nitrite to NH4) | nrfA (dissimilatory nitrite reductase) | Bacteria; wide taxonomic range | nrfA-F2aw/7R1 | 1.4 μM; Bio-Rad Sso | 40 cycles of 95 °C/5 s, 60 °C/30 s | 90% | *Anaeromyxobacter dehalogenan*s; CP001359.1 | Welsh et al., 2014 |
| Nitrogen cycling (denitrification (nitrite to NO) | nirk: copper-containing nitrite reductase | Bacteria | nirK-583-F/909-R | 0.6 μM; Bio-Rad Sso | 40 cycles of 95 °C/5 s, 60 °C/30 s | 98% | Bradyrhizobium sp. D209a; AB480457.1 | Liu et al., 2003 |
| Nitrogen cycling (denitrification (nitrite to NO) | A-nirk: nitrite reductase | Archaea | anirKa_58F/578R | 0.6 μM; BioLine SensiFAST | 45 cycles of 95 °C/5 s, 60 °C/30 s | 92% | Candidatus Nitrosotenuis cloacae strain SAT1; CP011097.2 | Lund et al., 2012 |
| Nitrogen cycling (denitrification (N2O to N2) | nosZ: nitrous oxide reductase | Bacteria: Clade I | nosZ2-F/R | 0.7 µM; Bio-Rad Itaq | 40 cycles of 95 °C/5 s, 60 °C/30 s | 94% | Chelatococcus daeguensis strain TAD1; JX394219.1 | Henry et al., 2006 |
| Phosphorous mineralization (Phosphoester and anhydride bond dephosphorylation) | phoD (alkaline phosphatase) | Bacteria; wide taxonomic range | phoD-733F/  R10831R | 1.5 μM; Bio-Rad Sso | 40 cycles of 95 °C/5 s, 60 °C/30 s | 85% | Gemmatimonas aurantiaca AP009153.1 | Ragot et al., 2015 |
| Phosphorous mineralization | phoC (acid phosphatase) | Bacteria; wide taxonomic range | phoC-A-F1/R1 | 0.5 μM; BioLine SensiFAST | 40 cycles of 95 °C/5 s, 60 °C/30 s | 95% | Cupriavidus oxalaticus CP032518.1;Ralstonia pickettii CP006668.1; Agrobacterium tumefaciens CP032928.1 | Fraser et al.,, 2017 |
| Phosphorous mineralization (Phosphoester and anhydride bond dephosphorylation) | phnX (Phosphono-acetaldehyde hydrolase) | Bacteria; Primarily Pseudomonadota Bacillota | phnX-FW/RW | 0.35 µM; Bio-Rad Itaq | 40 cycles of 95 °C/5 s, 60 °C/30 s | 86% | Rhodoferax sp. DCY110 CP019236.1;Acidovorax avenae CP028300.1;Variovorax sp. PMC12 CP027773.1;Rhodospirillaceae sp. ECT2AJA-110-B CP030885.1;Pseudomonas frederiksbergensis CP018319.1 | Bergkemper et al., 2016 |
| Phosphorous solubilization (C-P bond cleavage) | pqqC (pyrroloquinoline quinone) | Bacteria; Primarily Pseudomonadota (Alpha-, Beta-, Gamma), Verrucomicrobia, Actinomycetota | pqqC-R/R | 1.0 µM; Bio-Rad Itaq | 40 cycles of 95 °C/5 s, 60 °C/30 s | 94% | Mycobacterium dioxanotrophicus CP020809.1;Rubrivivax gelatinosus AP012320.1;Pseudonocardia sp. EC080619-01 CP012184.1;Streptomyces dengpaensis CP026652.1;Mycolicibacterium smegmatis KU678343.1;Pseudonocardia sp CP012184.1Pseudomonas psychrotolerans CP021645.1; | (Zheng et al., 2017) |

**a** Indicates general targeted function **b** All assays started with an initial denaturation step of 95 °C for 3 min and finished with a melt curve from 65 °C to 95 °C; **c** Excluding un-cultured references, Equimolar plasmid pools were used in assays where more than one organisms is listed. Sso: Sso Advanced™ Universal SYBR® Green Supermix (Bio-Rad, USA); Sensifast: SensiFAST™ Real-Time master mix (Bioline, UK); Itaq: Itaq Universal SYBR® Green Supermix (Bio-Rad, USA); all assays are run to extinction

**Additional quantitative PCR methods:**

To generate plasmid standards, soil DNA was amplified in 50 µL PCR reactions using the appropriate primers and GoTaq master mix (Promega, USA). PCR products were assessed on a 1% agarose gel to verify that a single amplicon of the correct size fragment was amplified. PCR products were then cleaned using either Omega Mag-Bind RxnPure Plus kits (Omega Bio-Tek, USA) or QIAquick PCR Purification Kit (Qiagen Inc., USA). The cleaned products were cloned, following the manufacturers protocol, into competent TOP10F’ One Shot E. coli (Invitrogen, USA). Clones were incubated overnight at 37°C on selective media (imMedia™ Growth Medium, Invitrogen), and positive clones were chosen and cultured in Luria Broth overnight at 37°C. Plasmids were extracted using either Wizard® Plus SV Minipreps DNA Purification Systems kit (Promega, USA) or QIAprep Spin Miniprep Kit (Qiagen Inc., USA). All plasmids were sequenced at AAC Genomics Facility (Guelph, Ontario) to confirm the plasmid insert identity.

**Quantitative PCR references:**

Bach HJ, Hartmann A, Schloter M, Munch JC. (2001). PCR primers and functional probes for amplification anddetection of bacterial genes for extracellular peptidases in single strains and in soil. J Microbiol Meth 44: 173–182.

Barbi F, Bragalini C, Vallon L, Prudent E, Dubost A, et al. (2014) PCR Primers to Study the Diversity of Expressed Fungal Genes Encoding Lignocellulolytic Enzymes in Soils Using High-Throughput Sequencing. PLOS ONE 9(12): e116264. doi: 10.1371/journal.pone.0116264

Bergkemper, F., Kublik, S., Lang, F., Krüger, J., Vestergaard, G., Schloter, M., Schulz, S. 2016. Novel oligonucleotide primers reveal a high diversity of microbes which drive phosphorous turnover in soil. Journal of Microbiological Methods, 125, pp. 91-97. doi: 10.1016/j.mimet.2016.04.011

Canizares, R, Benitez, E, Ogunseitan, OA. 2011. Molecular analysis of B-glucosidase diversity and function in soil. Eur. J. Soil Biol. 47: 1-8

Edwards, IP, Upchurch, RA, Zak, DR. 2008. Isolation of fungal cellobiohydrolase I genes from sporocarps and forest soils by PCR. Appl. Environ. Microbiol. 74: 3481-3489

Fraser, T.D., Lynch, D.H., Gaiero, J., Khosla, K., Dunfield, K.E., 2017. Quantification of bacterial non-specific acid (phoC) and alkaline (phoD) phosphatase genes in bulk and rhizosphere soil from organically managed soybean fields. Applied Soil Ecology 111, 48–56. http://dx.doi.org/10.1016/j.apsoil.2016.11.013.

Gregory, L.G., Karakas-Sen, A., Richardson, D.J. and Spiro, S. 2000. Detection of genes for membrane-bound nitrate reductase in nitrate-respiring bacteria and in community DNA. FEMS Microbiol. Lett. 183, 275–279

Henry, S., Bru, D., Stres, B., Hallet, S., and Philippot, L. (2006) Quantitative detection of the nosZ gene, encoding nitrous oxide reductase, and comparison of the abundances of 16S rRNA, narG, nirK, and nosZ genes in soils. Appl Environ Microbiol 72: 51

Kellner, H., Luis, P., Buscot, F., 2007a. Diversity of laccase-like multicopper oxidase (LMCO) genes in Morchellaceae: identification of genes potentially involved in extracellular activities related to plant litter decay. FEMS Microbiology Ecology 61, 153–163

Liu, X., S. M. Tiquia, G. Holguin, L. Wu, S. C. Nold, A. H. Devol, K. Luo, A. V. Palumbo, J. M. Tiedje, and J. Zhou. 2003. Molecular diversity of denitrifying genes in continental margin sediments within the oxygen-deficient zone off the Pacific coast of Mexico. Appl. Environ. Microbiol. 69:3549-3560.

Lund MB, Smith JM, Francis CA. 2012. Diversity, abundance and expression of nitrite reductase (nirK)-like genes in marine thaumarchaea. Isme 6: 1966-1977

Meinhardt, K. A., Bertagnolli, A., Pannu, M. W., Strand, S. E., Brown, S. L. and Stahl, D. A. (2015), Evaluation of revised polymerase chain reaction primers for more inclusive quantification of ammonia-oxidizing archaea and bacteria. Environmental Microbiology Reports, 7: 354–363. doi:10.1111/1758-2229.12259

Poly F,Wertz S, Brothier E & Degrange V (2008) First exploration of Nitrobacter diversity in soils using functional gene nxrA encoding nitrite oxido-reductase. FEMS Microbiol Ecol 63: 132–140.

Ragot, S. A., Kertesz, M. A., & Bünemann, E. K. (2015). phoD Alkaline Phosphatase Gene Diversity in Soil. Applied and Environmental Microbiology, 81(20), 7281–7289. http://doi.org/10.1128/AEM.01823-15

Raskin L, Stromley JM, Rittmann BE, Stahl DA (1994) Group-specific 16S rRNA hybridization probes to describe natural communities of methanogens. Appl Environ Microbiol 60: 1232–1240.e105592; Takai K, Horikoshi K (2000) Rapid detection and quantification of members of the archaeal community by quantitative PCR using fluorogenic probes. Appl Environ Microbiol 66: 5066–5072.https://doi.org/10.1371/journal.pone.0105592

Rotthauwe J-H, Witzel K-P, Liesack W. 1997. The ammonia monooxygenase structural gene amoA as a functional marker: molecular fine-scale analysis of natural ammonia-oxidizing populations. Applied Environ Microbiol 63:4704-4712

Vainio EJ, Hantula J. Direct analysis of wood-inhabiting fungi using denaturing gradient gel electrophoresis of amplified ribosomal DNA. Mycol Res. 2000;104:927–936

Watanabe, K., Y. Kodama, and S. Harayama. 2001. Design and evaluation of PCR primers to amplify bacterial 16S ribosomal DNA fragments used for community fingerprinting. J. Microbiol. Methods 44:253–262.

Welsh A, Chee-Sanford JC, Connor LM, Löffler FE, Sanford RA (2014) Refined NrfA phylogeny improves PCR-based nrfA gene detection. Appl EnvironMicrobiol 80(7):2110–2119.

Zheng, B-X., Hao, X., Ding, K., Zhou, G-W., Chen, Q-L., Zhang, J-B., & Zhu, Y-G. (2017). Long-term nitrogen fertilization decreased the abundance of inorganic phosphate solubilizing bacteria in an alkaline soil. ScientificReports, 7, [42284]. https://doi.org/10.1038/srep42284

**SUPPLEMENTARY METHODS: SEQUENCING ANALYSIS**

**Bioinformatics analyses:**

Sequence quality was assessed using FastQC (v. 0.11.5; Andrews, 2010), primers were removed using cutadapt (v. 1.15-0; Martin, 2011), paired-end reads were assembled using PEAR (uncalled base threshold set to 0, v. 0.9.10; Zhang et al. 2014), sequences were filtered using VSEARCH (max error of 1, v. 2.4.3; Rognes et al. 2016) and subsequently trimmed using Trimmomatic (truncation lengths set to 375 and 500 for the 16S and ITS2 datasets, respectfully; version 0.36; Bolger et al. 2014). Both datasets were inspected again using FastQC to ensure all read Phred quality scores were greater than 30. Dereplication, singleton removal, de novo chimera checking, and clustering at 98% sequence identity were carried out using VSEARCH. For ITS2 sequences, an ITS2 region extraction was performed using ITSx (version 1.0.11; Bengtsson-Palme et al., 2013) prior to clustering. For the 16S rRNA genes, taxonomy was assigned with the assign_taxonomy.py script in QIIME 1.9.1 (Caporaso et al., 2010) against the GreenGenes database (99% OTUS; version 13.8; DeSantis et al., 2006) using the Ribosomal Database Project (RDP) classifier (boot-strap confidence minimum of 0.8; Wang et al., 2007). ITS2 reads were classified similarly except against the UNITE database (version 8.2; dynamic reference file; Nilsson et al., 2019). OTU tables were generated in QIIME 1.9.1 and rarefaction was carried out using a sequence depth of 19,788 and 119,028.000 sequences per sample for the 16S and ITS datasets, respectively. Prior to downstream analysis, 16S and ITS OTU tables were filtered to remove chloroplasts/mitochondria and any non-fungal hits, respectively, using the filter_taxa_from_otu_table.py script in QIIME 1.9.1.

**Bioinformatics references:**

Andrews S. (2010). FastQC: a quality control tool for high throughput sequence data. Available online at: <http://www.bioinformatics.babraham.ac.uk/projects/fastqc>

Bengtsson‐Palme, J., Ryberg, M., Hartmann, M., Branco, S., Wang, Z., Godhe, A., … Nilsson, R.H.. (2013). Improved software detection and extraction of ITS1 and ITS2 from ribosomal ITS sequences of fungi and other eukaryotes for analysis of environmental sequencing data. *Methods in Ecology and Evolution*, *4*, 914-919 https://doi:10.1111/2041-210x.12073

Bolger, A. M., Lohse, M., & Usadel, B. (2014). Trimmomatic: a flexible trimmer for Illumina sequence data. *Bioinformatics,* *30*, 2114-2120. https://doi:10.1093/bioinformatics/btu170

Caporaso, J. G., Kuczynski, J., Stombaugh, J., Bittinger, K., Bushman, F. D., Costello, E. K., …Knight, R. (2010). QIIME allows analysis of high-throughput community sequencing data. *Nature Methods*, *7*, 335-336. https://doi:10.1038/nmeth.f.303

DeSantis, T. Z., Hugenholtz, P, Larsen, N., Rojas, M., Brodie, E. L., Keller, K., …Andersen, G.L. (2006). Greengenes, a chimera-checked 16S rRNA gene database and workbench compatible with ARB. *Applied and Environmental Microbiology,* *72,* 5069-5072. https://doi:10.1128/AEM.03006-05.

Martin, M. (2011). Cutadapt removes adapter sequences from high-through sequencing reads. *EMBnet.journal*, *17*, 10-12. https://doi:10.14806/ej.17.1.200

Nilsson, R. H., Larsson, K. H., Taylor, A. F. S., Bengtsson-Palme, J., Jeppesen, T. S., Schigel, D., … Abarenkov, K. (2018). The UNITE database for molecular identification of fungi: handling dark taxa and parallel taxonomic classifications. *Nucleic Acids Research*, *47*, D259-D264. https://doi:10.1093/nar/gky1022

Rognes, T., Flouri, T., Nichols, B., Quince, C., & Mahé, F. (2016). VSEARCH: a versatile open source tool for metagenomics. *PeerJ*, *4*, e2584. https://doi:10.7717/peerj.2584.

Wang, Q., Garrity, G. M., Tiedje, J. M., & Cole, J. R. (2007). Naїve Bayesian classifier for rapid assignment of rRNA sequences into the new bacterial taxonomy. *Applied and Environmental Microbiology*, *73*, 5261-5267. https://doi:10.1128/AEM.00062-07

Zhang, J., Kobert, K., Flouri, T., & Stamatakis, A. (2014). PEAR: a fast and accurate Illumina Paired-End reAd mergeR. *Bioinformatics,* *30*, 614-620. https://doi:10.1093/bioinformatics/btt593

Table S2. Sequence processing summary for Bacterial 16S and Fungal ITS amplicon sequencing

| **Processing step** | **Bacterial 16S - Dataset** | **Fungal ITS - Dataset** |
| --- | --- | --- |
| Sample Number | 60 | 60 |
| Read 1 | 23,676,439 | 17,760,536 |
| Read 2 | 23,676,439 | 17,760,536 |
| Primer removal/merging | 22,734,812 | 17,238,054 |
| Filtering (maxee=1) | 17,210,303 | 15,382,391 |
| Trimming (crop=375) | 17,210,303 | 15,382,391 |
| Total Sequence Loss (before clustering) | 6,466,136 | 2,378,145 |
| Cumulative % Sequence Loss (before clustering) | 27.31% | 13.39% |
| Remaining Sequences (after clustering) | 12,843,763 | 14,478,951 |
| Cumulative % Sequence Loss (after clustering) | 45.75% | 18.48% |
| OTU Count (non-rarefied) \| 98% | 32,013 | 16,477 |
| Min | 19,788.00 | 119,028.00 |
| Max | 257,026.00 | 305,405.00 |
| Median | 189,397.50 | 201,367.00 |
| Mean | 183,482.33 | 206,842.16 |

Table S3. Two-way ANOVA analysis: Quantitative PCR of taxonomic groups and nitrogen, carbon, phosphorous cycling processes, calculated as gene copies ng^-1^ DNA and averaged across both sampling depths.

|  | Time | | | | 2-way ANOVA p values | | |
| --- | --- | --- | --- | --- | --- | --- | --- |
| Target Gene | Forest | 1-10yrs | 11-50yrs | >50yrs | Time | Depth^†^ | Time:Depth |
| B16S (*x10^6^*) | 1.92 (0.22) | 2.19 (0.39) | 2.13 (0.20) | 2.02 (0.24) | 0.06 | 0.89 | 0.915 |
| A16S (*x10^3^*) | **3.93 (1.67)c** | **8.07 (4.53)ab** | **5.51 (1.34)bc** | **9.51 (3.84)a** | **<0.001** | 0.37 | 0.646 |
| F18S (*x10^4^*) | **1.62 (0.53)b** | **3.32 (1.15)a** | **2.68 (1.00)a** | **3.28 (1.14)a** | **<0.001** | **<0.001^a^** | 0.693 |
| Laccase (*x10^4^*) | 2.19 (0.67) | 2.20 (0.39) | 2.41 (0.44) | 2.14 (0.56) | 0.59 | 0.42 | 0.338 |
| Cbh (*x10*) | **1.33 (0.29)b** | **2.43 (1.11)a** | **1.69 (0.47)ab** | **1.81 (0.67)ab** | **<0.001** | **<0.01^a^** | 0.472 |
| B-glu (*x10^3^*) | **2.35 (1.18)b** | **2.55 (0.91)ab** | **2.27 (0.38)ab** | **3.10 (1.10)a** | **0.04** | **0.02^b^** | 0.372 |
| Gh11 (*x10*) | **4.13 (0.94)b** | **8.66 (2.92)a** | **7.73 (2.59)a** | **7.67 (2.56)a** | **<0.001** | **<0.001^a^** | 0.838 |
| APR (*x10^3^*) | 7.29 (1.57) | 6.31 (1.19) | 6.61 (1.10) | 6.35 (1.74) | 0.15 | 0.19 | 0.831 |
| B-amoA (*x10^2^*) | **0.73 (0.73)b** | **6.13 (5.41)a** | **3.79 (2.41)a** | **6.48 (6.58)a** | **<0.001** | 0.15 | 0.201 |
| A-amoA (*x10^2^*) | **3.42 (2.96)b** | **7.61 (7.12)a** | **8.24 (3.29)a** | **9.03 (3.42)a** | **<0.001** | **0.03^b^** | 0.698 |
| nxrA (*x10*) | **0.97 (1.33)b** | **12.63 (9.32)a** | **5.91 (3.98)a** | **10.27 (5.98)a** | **<0.001** | 0.18 | 0.670 |
| narG (*x10^5^*) | 3.56 (0.63) | 3.30 (0.41) | 3.44 (0.49) | 3.44 (0.40) | 0.57 | 0.11 | 0.904 |
| Nrf (*x10^3^*) | 8.04 (2.76) | 6.27 (1.95) | 6.60 (1.48) | 7.74 (2.51) | 0.14 | 0.67 | 0.950 |
| nirK (*x10^4^*) | 3.85 (0.65) | 3.73 (0.87) | 3.98 (0.64) | 3.66 (0.63) | 0.70 | 0.11 | 0.825 |
| aNirK (*x10^2^*) | **0.51 (0.12)ab** | **0.44 (0.13)b** | **0.70 (0.59)ab** | **0.94 (1.25)a** | **<0.01** | **0.03^b^** | 0.089 |
| nosZ (*x10^3^*) | 5.83 (1.13) | 5.88 (1.43) | 5.45 (0.72) | 6.26 (1.21) | 0.46 | 0.94 | 0.932 |
| phoD (*x10^5^*) | **2.38 (0.63)ab** | **1.83 (0.37)b** | **2.02 (0.34)ab** | **1.91 (0.36)b** | **<0.01** | 0.15 | 0.825 |
| phoC (*x10^3^*) | **6.97 (1.43)a** | **5.28 (0.93)b** | **4.98 (1.10)b** | **5.13 (0.91)b** | **<0.001** | 0.09 | 0.329 |
| phnX (*x10^3^*) | 1.46 (0.32) | 1.45 (0.34) | 1.33 (0.31) | 1.69 (0.45) | 0.11 | 0.33 | 0.610 |
| pqqC (*x10^4^*) | 1.13 (0.21) | 1.15 (0.2) | 1.03 (0.19) | 1.07 (0.11) | 0.32 | 0.80 | 0.852 |

Data are presented as means averaged across depths for each time grouping, with standard deviation in parentheses; all copy number values are at the scientific notation indicated next to the gene name. Letters within a row indicate significant differences between time groups across depth increment, based on the factor “time” 2-way ANOVA p value. ^†^When significant differences occurred with depth (averaged across all time groups), differences are indicated as follows: **^a^**gene abundances higher in the 0-5cm and **^b^**gene abundances higher in the 5-15cm depth. Full information on functions and groups underlying the gene abbreviations may be found in Supplementary Table 1.

Table S4. Two-way ANOVA analysis: Quantitative PCR of taxonomic groups and nitrogen, carbon, phosphorous cycling processes, calculated as gene copies g^-1^ dry soil and averaged across both sampling depths.

|  | Time | | | | 2-way ANOVA p values | | |
| --- | --- | --- | --- | --- | --- | --- | --- |
| Target Gene | Forest | 1-10yrs | 11-50yrs | >50yrs | Time | Depth^†^ | Time:Depth |
| B16S (*x10^10^*) | 5.65 (2.38) | 4.47 (1.14) | 5.00 (1.67) | 4.47 (1.99) | 0.46 | 0.21 | 0.98 |
| A16S (*x10^8^*) | 1.20 (0.85) | 1.72 (1.11) | 1.24 (0.34) | 2.33 (1.80) | 0.08 | 0.16 | 0.95 |
| F18S (*x10^8^*) | **4.80 (2.56)b** | **6.77 (2.6)a** | **6.18 (2.69)a** | **7.56 (4.85)a** | **0.05** | **<0.001** | 0.93 |
| Laccase (*x10^8^*) | 4.52 (2.25) | 2.76 (0.89) | 3.33 (1.23) | 3.14 (1.43) | 0.11 | 0.74 | 0.75 |
| Cbh (*x10^5^*) | 4.08 (2.23) | 5.20 (3.73) | 3.88 (1.28) | 4.12 (2.57) | 0.53 | **<0.01** | 0.89 |
| B-glu (*x10^7^*) | 6.72 (3.99) | 5.35 (2.15) | 5.26 (1.58) | 6.57 (2.93) | 0.62 | 0.66 | 0.83 |
| Gh11 (*x10^6^*) | 1.26 (0.71) | 1.80 (0.75) | 1.79 (0.73) | 1.84 (1.47) | 0.08 | **<0.001** | 1.00 |
| APR (*x10^8^*) | 2.22 (1.11) | 1.33 (0.51) | 1.55 (0.57) | 1.47 (0.92) | 0.08 | 0.61 | 0.92 |
| B-amoA (*x10^7^*) | **0.23 (0.24)b** | **1.29 (1.05)a** | **0.80 (0.42)a** | **1.44 (1.38)a** | **<0.001** | 0.08 | 0.48 |
| A-amoA (*x10^7^*) | **1.06 (1.17)b** | **1.65 (1.73)a** | **1.83 (0.64)ab** | **1.97 (0.99)a** | **<0.01** | 0.19 | 0.88 |
| nxrA (*x10^6^*) | **0.34 (0.56)b** | **2.49 (1.67)a** | **1.28 (0.83)a** | **2.44 (2.48)a** | **<0.001** | 0.10 | 0.81 |
| narG (*x10^9^*) | 10.48 (4.47) | 6.93 (2.30) | 8.10 (3.02) | 7.59 (3.18) | 0.12 | 0.49 | 0.92 |
| Nrf (*x10^8^*) | **2.28 (1.04)a** | **1.34 (0.61)ab** | **1.51 (0.44)b** | **1.73 (0.87)ab** | **0.05** | 0.19 | 0.96 |
| nirK (*x10^8^*) | 11.56 (5.49) | 7.85 (3.10) | 9.38 (3.64) | 8.17 (3.66) | 0.20 | 0.75 | 0.88 |
| aNirK (*x10^6^*) | 1.57 (0.81) | 0.93 (0.38) | 1.45 (0.98) | 1.76 (1.80) | 0.07 | 0.40 | 0.10 |
| nosZ (*x10^8^*) | 1.76 (0.86) | 1.26 (0.56) | 1.28 (0.44) | 1.42 (0.76) | 0.43 | 0.32 | 0.99 |
| phoD (*x10^9^*) | **7.03 (3.26)ab** | **3.79 (1.25)ab** | **4.84 (2.200)b** | **4.24 (1.84)b** | **<0.01** | 0.60 | 0.87 |
| phoC (*x10^8^*) | **2.15 (1.24)ab** | **1.11 (0.41)ab** | **1.13 (0.31)b** | **1.15 (0.57)b** | **<0.001** | 0.67 | 0.72 |
| phnX (*x10^7^*) | 4.45 (2.27) | 3.08 (1.38) | 3.15 (1.43) | 3.74 (2.02) | 0.36 | 0.13 | 0.96 |
| pqqC (*x10^8^*) | 3.41 (1.67) | 2.40 (0.82) | 2.45 (1.01) | 2.37 (1.06) | 0.20 | 0.21 | 0.94 |

Data are presented as means averaged across depths for each time grouping, with standard deviation in parentheses; all copy number values are at the scientific notation indicated next to the gene name. Letters within a row indicate significant differences between time groups across depth increment, based on the factor “time” 2-way ANOVA p value. ^†^Where significant differences occurred with depth, gene abundances were higher in the 0-5cm. Full information on functions and groups underlying the gene abbreviations may be found in Supplementary Table 1.

Table S5. The relative abundance of different taxonomic groups and nitrogen, carbon, phosphorous cycling processes, shown as gene copies g^-1^ dry soil for each depth increment. One-way ANOVA analysis with time as the main factor

|  | 0-5cm; gene copies g-1 dry soil | | | | | 5-15cm; gene copies g-1 dry soil | | | | |
| --- | --- | --- | --- | --- | --- | --- | --- | --- | --- | --- |
| Target gene | Forest | 1-10yrs | 11-50yrs | >50yrs | p value | Forest | 1-10yrs | 11-50yrs | >50yrs | p value |
| B16S (*x10^10^*) | 6.25 (2.82) | 4.74 (1.19) | 5.37 (2.01) | 4.71 (2.34) | 0.45 | 5.06 (1.82) | 4.20 (1.09) | 4.63 (1.38) | 4.24 (1.69) | 0.61 |
| A16S (*x10^8^*) | 1.37 (1.10) | 2.00 (1.37) | 1.34 (0.38) | 2.87 (2.22) | 0.18 | 1.03 (0.50) | 1.44 (0.75) | 1.15 (0.31) | 1.79 (1.17) | 0.24 |
| F18S (*x10^8^*) | 6.18 (2.69) | 7.92 (2.00) | 7.98 (2.60) | 9.77 (5.65) | 0.26 | 3.42 (1.54) | 5.61 (2.73) | 4.38 (1.19) | 5.35 (2.70) | 0.19 |
| Laccase (*x10^8^*) | **5.13 (2.66)a** | **2.71 (0.88)b** | **3.39 (1.38)ab** | **3.00 (1.50)ab** | **0.04** | 3.92 (1.68) | 2.81 (0.95) | 3.27 (1.22) | 3.28 (1.44) | 0.44 |
| Cbh (*x10^5^*) | 4.99 (2.49) | 5.23 (2.04) | 4.61 (1.03) | 5.20 (3.06) | 0.97 | 3.18 (1.58) | 5.17 (5.07) | 3.15 (1.13) | 3.04 (1.48) | 0.79 |
| B-glu (*x10^7^*) | 6.03 (2.69) | 5.53 (2.00) | 5.13 (0.97) | 6.20 (3.17) | 0.86 | 7.42 (5.05) | 5.16 (2.42) | 5.39 (2.16) | 6.94 (2.83) | 0.62 |
| Gh11 (*x10^6^*) | 1.56 (0.86) | 2.09 (0.54) | 2.19 (0.87) | 2.30 (1.87) | 0.43 | 0.97 (0.38) | 1.51 (0.84) | 1.39 (0.19) | 1.38 (0.83) | 0.33 |
| APR (*x10^8^*) | 2.42 (1.17) | 1.35 (0.49) | 1.63 (0.66) | 1.37 (0.70) | 0.04 | 2.02 (1.09) | 1.32 (0.57) | 1.48 (0.52) | 1.56 (1.13) | 0.32 |
| BamoA (*x10^7^*) | **0.19 (0.15)b** | **1.85 (1.24)a** | **1.03 (0.52)a** | **1.91 (1.81)a** | **<0.001** | **0.27 (0.31)b** | **0.74 (0.36)ab** | **0.58 (0.09)ab** | **0.97 (0.56)a** | **<0.001** |
| AamoA (*x10^7^*) | 1.01 (1.44) | 1.67 (1.80) | 1.70 (0.59) | 1.70 (0.87) | 0.16 | 1.12 (0.92) | 1.64 (1.79) | 1.96 (0.74) | 2.24 (1.09) | 0.11 |
| nxrA (*x10^6^*) | **0.36 (0.65)b** | **3.38 (1.57)a** | **1.64 (0.96)a** | **2.79 (3.06)a** | **<0.01** | **0.32 (0.49)b** | **1.60 (1.31)a** | **0.92 (0.55)a** | **2.10 (1.88)a** | **<0.01** |
| narG (*x10^9^*) | 11.30 (5.05) | 7.36 (2.46) | 8.03 (3.36) | 7.70 (3.68) | 0.15 | 9.62 (3.91) | 6.50 (2.20) | 8.18 (3.04) | 7.48 (2.86) | 0.23 |
| Nrf (*x10^8^*) | 2.50 (1.02) | 1.41 (0.64) | 1.65 (0.49) | 1.83 (0.94) | 0.07 | 2.06 (1.07) | 1.27 (0.61) | 1.37 (0.40) | 1.62 (0.85) | 0.24 |
| nirK (*x10^8^*) | 12.80 (6.49) | 8.03 (3.32) | 9.67 (4.72) | 8.06 (4.27) | 0.17 | 10.30 (4.29) | 7.68 (3.08) | 9.09 (2.70) | 8.28 (3.23) | 0.45 |
| aNirK (*x10^6^*) | **1.83 (0.87)a** | **0.88 (0.33)b** | **1.02 (0.44)ab** | **1.16 (0.49)ab** | **0.02** | 1.30 (0.68) | 0.97 (0.45) | 1.88 (1.23) | 2.35 (2.43) | 0.14 |
| nosZ (*x10^8^*) | 1.95 (1.02) | 1.29 (0.46) | 1.37 (0.51) | 1.55 (0.95) | 0.39 | 1.58 (0.66) | 1.22 (0.67) | 1.19 (0.40) | 1.30 (0.53) | 0.56 |
| phoD (*x10^9^*) | **7.52 (3.32)a** | **3.98 (1.30)b** | **4.90 (2.38)ab** | **4.08 (1.97)b** | **0.02** | 6.53 (3.32) | 3.59 (1.25) | 4.79 (2.29) | 4.40 (1.83) | 0.09 |
| phoC (*x10^8^*) | **2.47 (1.49)a** | **1.09 (0.38)b** | **1.08 (0.36)b** | **1.12 (0.54)b** | **0.05** | 1.83 (0.89) | 1.12 (0.46) | 1.18 (0.29) | 1.19 (0.64) | 0.11 |
| phnX (*x10^7^*) | 4.95 (2.61) | 3.33 (1.28) | 3.73 (1.78) | 4.02 (2.39) | 0.47 | 3.95 (1.88) | 2.83 (1.53) | 2.56 (0.75) | 3.46 (1.67) | 0.37 |
| pqqC (*x10^8^*) | 3.89 (1.99) | 2.63 (0.85) | 2.63 (1.25) | 2.45 (1.26) | 0.17 | 2.94 (1.22) | 2.17 (0.77) | 2.27 (0.81) | 2.30 (0.90) | 0.37 |

Quantitative PCR data are presented as means with standard deviation in parentheses; all copy number values are at the scientific notation indicated with the gene name. Full information on functions and groups underlying the gene abbreviations may be found in Supplementary Table 1. Letters indicate significant differences between time groups for each separate depth increment, based on the provided p value for the one-way ANOVA .

Table S6: PERMANOVA outcome associated with NMDS clusters

| **Time increment** | **>50yrs** | **1-10yrs** | **11-50yrs** | **>50yrs** | **1-10yrs** | **11-50yrs** |
| --- | --- | --- | --- | --- | --- | --- |
| **Group** | **Bacterial 16S rRNA** | | | **Fungal ITS** | | |
|  | **Across both depths** | | | | | |
| **1-10yrs** | **0.025** |  |  | **0.003** |  |  |
| **11-50yrs** | **0.018** | 0.116 |  | **0.027** | **0.027** |  |
| **Forest** | **0.002** | **0.002** | **0.002** | **0.002** | **0.002** | **0.002** |
|  | **5 cm depth increment** | | | | | |
| **1-10yrs** | 0.185 |  |  | 0.179 |  |  |
| **11-50yrs** | 0.182 | 0.486 |  | 0.509 | 0.509 |  |
| **Forest** | **0.002** | **0.002** | **0.002** | **0.003** | **0.004** | **0.003** |
|  | **15 cm depth increment** | | | | | |
| **1-10yrs** | 0.350 |  |  | 0.129 |  |  |
| **11-50yrs** | 0.350 | 0.596 |  | 0.163 | 0.363 |  |
| **Forest** | **0.042** | **0.042** | 0.102 | **0.006** | **0.006** | **0.006** |

Table S7. Physical and chemical soil properties.

| **Time group** | **Forest** | **1-10yrs** | **11-50yrs** | **>50yrs** |
| --- | --- | --- | --- | --- |
| **Depth: 0-5 cm** | | | | |
| pH | 6.05 (0.37) | 5.73 (0.56) | 6.14 (0.30) | 6.09 (0.31) |
| **P (mg kg^-1^) *** | **5.7 (2.1) b** | **8.3 (5.4) ab** | **13.1 (12.9) ab** | **11.1 (6.9) a** |
| K (mg kg^-1^) | 187 (90) | 172 (59) | 231 (100) | 218 (147) |
| Mg (mg kg^-1^) | 879 (463) | 466 (297) | 474 (203) | 650 (210) |
| Fe (mg kg^-1^) | 15.1 (12.2) | 16.2 (4.5) | 10.6 (3.8) | 17.4 (22.1) |
| Mn (mg kg^-1^) | 15.8 (5.9) | 12.3 (3.8) | 10.9 (5.6) | 9.6 (7.7) |
| Zn (mg kg^-1^) | 1.7 (1.3) | 2.0 (1.4) | 2.6 (1.7) | 1.3 (0.7) |
| **Total N (mg kg^-1^)**** | **0.67 (0.19) a** | **0.32 (0.12) b** | **0.43 (0.22) b** | **0.34 (0.12) b** |
| **Total C (mg kg^-1^)**** | **9.96 (3.67) a** | **4.08 (1.38) b** | **5.59 (3.48) b** | **3.74 (1.08) b** |
| Sand (%) | 14.3 (14.7) | 20.8 (24.7) | 6.10 (3.00) | 18.9 (16.3) |
| Silt (%) | 56.0 (9.5) | 53.1 (17.2) | 62.9 (11.0) | 56.8 (14.4) |
| Clay (%) | 29.7 (13.7) | 26.1 (12.5) | 30.9 (9.6) | 24.3 (7.4) |
| **Organic Matter (%) **** | **15.1 (6.7) a** | **5.1 (2.1) b** | **5.7 (3.0) b** | **7.9 (4.2) b** |
| **Depth: 5-15cm** | | | | |
| pH | 6.25 (0.38) | 5.88 (0.41) | 6.34 (0.31) | 6.3 (0.34) |
| P (mg kg -1) | 2.7 (1.1) | 3.8 (1.0) | 7.6 (7.7) | 5.0 (3.3) |
| K (mg kg -1) | 121 (64) | 100 (35) | 149 (73) | 132 (100) |
| Mg (mg kg -1) | 716 (378) | 431 (298) | 488 (246) | 683 (230) |
| Fe (mg kg -1) | 24.8 (16.4) | 18.9 (6.3) | 11.3 (4.8) | 14.9 (13.6) |
| Mn (mg kg -1) | 8.9 (4.1) | 8.5 (2.1) | 7.5 (5.5) | 5.5 (3.4) |
| Zn (mg kg -1) | 1.1 (1.1) | 1.1 (0.6) | 1.5 (1.2) | 0.8 (0.4) |
| **Total N (mg kg -1)*** | **0.35 (0.13) a** | **0.23 (0.08) ab** | **0.33 (0.18) ab** | **0.23 (0.04) b** |
| **Total C (mg kg -1)*** | **5.18 (2.01) a** | **3.05 (1.00) b** | **4.29 (2.78) b** | **2.67 (0.27) b** |
| **Sand (%) *** | **20.1 (19.7) a** | **22.2 (26.6) a** | **7.1 (3.8) b** | **22.2 (18.4) a** |
| Silt (%) | 51.3 (14.1) | 52.4 (18.5) | 62.8 (13.4) | 53.9 (13.4) |
| Clay (%) | 28.6 (12.1) | 25.4 (13.8) | 30.1 (10.0) | 23.9 (9.3) |
| **Organic Matter (%) **** | **7.5 (3.6) a** | **4.9 (1.2) b** | **4.2 (0.8) b** | **7.1 (4.7) b** |

Data are presented as means with standard deviation in parentheses. Data from Benalcazar et al. (2022) was used to explore the relationship between biological properties and soil abiotic properties. Letters indicate significant differences between time groups for each separate depth increment, based on the provided significance level from one-way ANOVA; * p < 0.05, ** p < 0.01 .
